# CO-free, aqueous mediated, instant and selective reduction of nitrobenzene *via* robustly stable chalcogen stabilised iron carbonyl clusters (Fe_3_E_2_(CO)_9_, E = S, Se, Te)†

**DOI:** 10.1039/d0ra04491a

**Published:** 2020-09-01

**Authors:** Charu Sharma, Avinash Kumar Srivastava, Aditi Soni, Sangeeta Kumari, Raj Kumar Joshi

**Affiliations:** Department of Chemistry, Malaviya National Institute of Technology Jaipur 302017 Rajasthan India rkjoshi.chy@mnit.ac.in

## Abstract

Highly stable and thermally robust iron chalcogenide carbonyl clusters Fe_3_E_2_(CO)_9_ (E = S, Se or Te) have been explored for the reduction of nitrobenzene. A 15 min thermal heating of an aqueous solution of nitrobenzene and hydrazine hydrate in the catalytic presence of Fe_3_E_2_(CO)_9_ (E = S, Se or Te) clusters yield average to excellent aniline transformations. Among the S, Se and Te based iron chalcogenised carbonyl clusters, the diselenide cluster was found to be most efficient and produce almost 90% yield of the desired amino product, the disulfide cluster was also found to be significantly active, produce the 85% yield of amino product, while the ditelluride cluster was not found to be active and produced only 49% yield of the desired product. The catalyst can be reused up to three catalytic cycles and it needs to be dried in an oven for one hour prior to reuse for the best results. The developed method is inexpensive, environmentally benign, does not require any precious metal or a high pressure of toxic CO gas and exclusively brings the selective reduction of the nitro group under feasible and inert free conditions.

## Abstract 2

In this study, a strongly feasible method for the reduction of nitrobenzene has been developed through highly stable and thermally robust iron chalcogenide carbonyl clusters Fe_3_E_2_(CO)_9_ (E = S, Se or Te).

## Graphical abstract

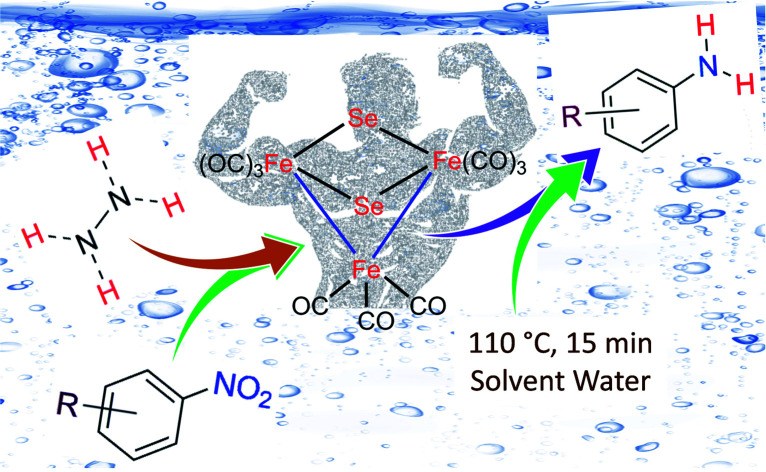

## Introduction

Since the initial application of an iron catalyst by Reppe and Vetter^1^ in 1953, iron-based catalysis has influenced the establishment of various catalysts based on 4d and 5d transition elements. However, before Reppe and Vetter's pioneering work, Fe in the form of iron carbonyl had been known since 1891.^2,3^ Being the second most abundant metal in the earth's crust, Fe is always under the limelight to explore as a catalyst for a variety of organic reactions. Since, an efficient, cost-effective, fast, clean and selective catalyst is needed in modern research, iron is the most attractive option available in the surroundings.^4^ However, catalysts derived from 4d and 5d transition metals and other rare earth metals are more efficient than 3d transition metals, but also expensive in meeting present and upcoming societal demands.^4^ Zero-valent iron in the form of Fe(CO)_5_ is one of the least expensive forms and of ubiquitous availability. *In situ* activation and catalysis by Fe(CO)_5_ require a strong base, moreover, the di-sodium tetracarbonyl ferrate Na_2_Fe(CO)_4_ and iron tetra carbonyl hydride H_2_Fe(CO)_4_ species were formed during the reaction, these species are highly temperature and light sensitive and drastically affect the productivity of the reaction.^5^ Nevertheless, due to the high catalytic potential of Fe(CO)_5_, it has been extensively used for various carbonylation reactions as well as as for high-nuclearity clustere formations.^6–11^ Electron-transfer,^12^ hydrogenation,^13^ hydride transfer^14^ and electrochemical methods^15^ are some of the important available methodologies to furnish distinct amines by utilizing a nitro precursor. The reduction of nitrobenzene has gained tremendous interest due to its hazardous effect on the environment. The process of reduction is quite complicated as the nitro group reduction advances in stages and stops due to the formation of hydroxylamine and azoarene as side products at an intermediate stage.^16^ Traditionally, catalytic transfer hydrogenation has been employed in the presence of Ru,^17^ Rh,^18^ Pd,^19^ or Ni (ref. 20) metal catalysts. Very few studies have utilized inexpensive Co,^21^ Fe,^22^ or Zn (ref. 23) metals for transfer hydrogenation; however, economic consequences strongly favour the iron-based system due to its greater environmental sustainability.

**Scheme 1:**
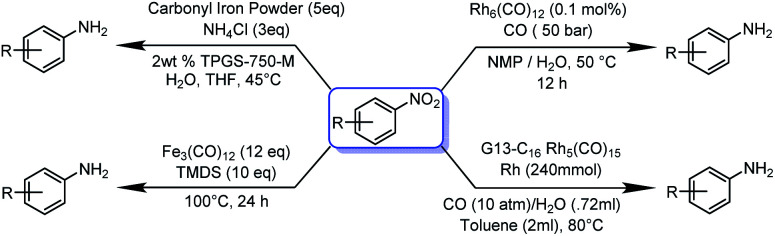
Previous reports on metal carbonyl catalysed nitro reduction reactions. Most metal carbonyl catalysed nitro reduction reactions require a high pressure of CO and H_2(g)_.

Traditionally, a plentiful inexpensive iron powder was used for nitro reduction, but it has low efficiency and produces a large amount of wastage. The nano-form of zero-valent iron (nZVI) was used for the reduction of nitro derivatives, but it was unsuccessful, as the catalytic activity of NPs is significantly related to their specific morphology, which is difficult to produce in bulk. Moreover, the formation of nZVI is a dynamic, time-consuming and laborious process. Apart from that, the accumulation of nZVI reduces its mobility, dispersibility and simultaneously reduces its catalytic reactivity.^24^ Zero-valent metal carbonyl clusters have some unique inherent properties of catalysis and thermal stability. Moreover, the absence of large bulk phases results in a high surface to volume ratio, which is beneficial for catalysis, since it minimizes the reaction rate per unit amount of catalyst, which likewise limits the cost.^25^ Metal carbonyls and clusters have been used for a wide range of catalysis: in particular, for carbonylation,^26^ C–C coupling,^27^ hydroformylation,^28^ hydroesterification,^29^ reduction of nitroaranes^30–32^ and many more value-added organic transformations.^6–11^ Initially (Scheme 1), Pittman and co-workers in 1979 utilised Rh_6_(CO)_12_ for nitro reduction.^33^ Later, in 2014, Kaneda and co-workers used a composite dendrimer of Rh_5_ carbonyl cluster and polyamine composite for nitro reduction.^34^ Recently in 2017, Lipshutz and co-workers used carbonyl iron powder under aqueous micellar catalysis condition.^35^ Fe(CO)_5_ and Fe_3_(CO)_12_ have also been used for nitro reduction, but all the methods require rather drastic reaction condition, including long duration, high pressure of CO gas, handling of highly toxic Fe(CO)_5_, reducing agent and various supporting reagents for to promote the reaction.^36^

Recently, chalcogen and metal chalcogenide complexes have come under the spotlight, these complexes are continuously breaking the stereotype of air and moisture sensitive metal complexes. Due to good electron donor properties of chalcogen atoms, chalcogenide complexes have emerged as a potential alternative to phosphine complexes. Their ability to withstand air/moisture and their permanence during catalysis makes them suitable catalysts for long-duration reactions, which include C–C couplings^37,38^ C–N couplings^39^ and other organic transformations.^40^

In addition to gaseous hydrogen, several other reducing agents have been used that allow the efficient reduction of nitrobenzene when used in combination with metals, including boranes,^41^ NaBH_4_,^42^ silanes^43^ and hydrazine.^44–46^ Hydrazine hydrate is used as a very suitable reducing reagent as it generates only N_2_ as a by-product. It is fairly safe and easy to handle compared with its unstable anhydrous form. Many nitro reduction protocols using a combination of iron complexes and hydrazine hydrate have been published in the last few years.^47–49^

**Scheme 2:**
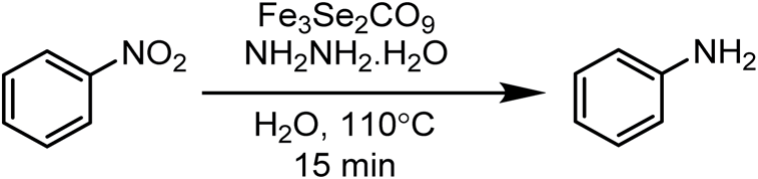
Nitro reductions catalysed by an Fe_3_Se_2_(CO)_9_ cluster.

This paper is designed to report a selective reduction of nitroarenes with hydrazine, catalysed by zero-valent Fe_3_Se_2_(CO)_9_ and Fe_3_S_2_(CO)_9_ carbonyl clusters. These clusters are robustly stable, insensitive towards air, moisture, water and sunlight, can be stored for a long time and can be easily synthesized on a large scale. The present method produces significant transformations and does not generate any waste (Scheme 2). The catalyst is found to be efficient and brings the desired transformation in just 15 min. Moreover, it works smoothly in aqueous media at 110 °C and is applicable for a large variety of functionalised nitrobenzenes.

## Results and discussion

Iron complexes have been established as excellent catalysts for various organic transformations, our group has also exploited quite a few iron based organic transformations.^6,47^ The literature encouraged us to investigate the catalytic activity of chalcogen-stabilized iron carbonyl complexes. This is the first report which utilises Fe_3_E_2_(CO)_9_ (E = S, Se or Te) clusters for the reduction of nitrobenzene and its derivatives to their corresponding arylamines. In the very first reaction, a dioxane solution of nitrobenzene and hydrazine (a source of hydrogen) with an Fe_3_Se_2_(CO)_9_ (3 mol%) cluster was refluxed with continuous monitoring of the reaction on a TLC. After 6 h, complete consumption of reactant along with the significant formation of the desired reduction product was experienced. The reaction was reinvestigated in the absence of Fe-catalyst, but no chemical transformation was recorded. Then, the reaction was optimized in search of the best suitable parameters for maximum transformation (Table 1). Initially, the amount of iron catalyst was optimised: 1 mol% of the catalyst failed to initiate the reaction, while 1.5 mol% of the catalyst started to produce the desire product in below to average amount (29%), however, the yield was improved from 29 to 47, 68 and 89% with 2, 2.5 and 3 mol% of the Fe-catalyst, respectively. No substantial improvement in the yield of aniline was experienced with 3.5 or 4 mol% of catalyst loading. Hence, 3 mol% of catalyst was the ideal amount for a creditable transformation (Table 1, entries 2–8). The other chalcogen-stabilised iron carbonyl clusters Fe_3_S_2_(CO)_9_ and Fe_3_Te_2_(CO)_9_ were also explored for the present reaction (Table 1, entries 9 and 10). It was observed that Fe_3_S_2_(CO)_9_ shows almost comparable catalytic efficiency to the selenium analogue, (82% yield of aniline), while the Fe_3_Te_2_(CO)_9_ was not efficient as it yielded average (49%) transformation of the desire product. Hence, the order of catalytic efficiency for these catalysts is Fe_3_Se_2_(CO)_9_ ≈ Fe_3_S_2_(CO)_9_ > Fe_3_Te_2_(CO)_9_. The difference in catalytic efficiency may be due to the electron-donor properties of S/Se/Te.^37^

**Table 1:** Optimization of various parameters with nitrobenzene

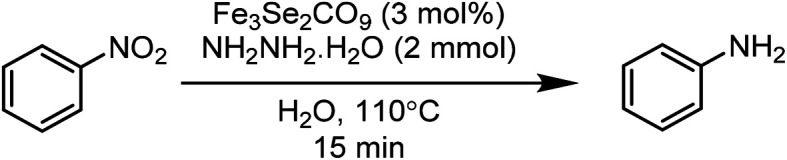							
S. no.	Solvent	Fe cat	Cat, mol%	N_2_H_4_, mmol	Temp., °C	Time (min)	Yield^a^, %
1	H_2_O	—	—	2	110	15	nd
2	H_2_O	**b**	1	2	110	15	nd
3	H_2_O	**b**	1.5	2	110	15	29
4	H_2_O	**b**	2	2	110	15	47
5	H_2_O	**b**	2.5	2	110	15	68
6	H_2_O	**b**	3	2	110	15	89
7	H_2_O	**b**	3.5	2	110	15	90
8	H_2_O	**b**	4	2	110	15	90
9	H_2_O	**a**	3	2	110	15	85
10	H_2_O	**c**	3	2	110	15	49
11	H_2_O	**b**	3	0.5	110	15	19
12	H_2_O	**b**	3	1	110	15	37
13	H_2_O	**b**	3	1.5	110	15	69
14	H_2_O	**b**	3	2	110	15	87
15	H_2_O	**b**	3	2.5	110	15	88
16	H_2_O	**b**	3	3.0	110	15	90
17	H_2_O	**b**	3	2	80	360	85
18	H_2_O	**b**	3	2	120	10	88
19	H_2_O	**b**	3	2	130	7	87
20	H_2_O	**b**	3	2	150	2	89
21	2-Propanol	**b**	3	2	110	15	27
22	Butanol	**b**	3	2	110	15	75
23	*sec*-Butanol	**b**	3	2	110	15	69
24	EtOH	**b**	3	2	110	15	79
	MeOH	**b**	3	2	110	15	82
25	Toluene	**b**	3	2	110	15	45
26	Dimethyl formamide	**b**	3	2	110	15	5
27	Dioxane	**b**	3	2	110	15	19

a Isolated yields, (**a**) Fe_3_S_2_(CO)_9_, (**b**) Fe_3_Se_2_(CO)_9_, (**c**) Fe_3_Te_2_(CO)_9_, optimised conditions: nitrobenzene (1 mmol), N_2_H_4_·H_2_O (2 mmol), catalyst Fe_3_Se_2_(CO)_9_ (3 mol%), temperature 110 °C, time 15 min, solvent water.

In a further attempt to optimize the amount of hydrazine hydrate which was used as a hydrogen source in the reaction, a 0.5 mmol amount of hydrazine produced 19% yield of aniline, while increasing the amount of hydrazine significantly increased the yield of aniline; however, no considerable improvement was observed beyond the 2 mmol amount of hydrazine (Table 1, entries 11–16). Here, 2 mmol of hydrazine hydrate serves as the optimal amount to achieve the desired quantity of hydrogen for the reaction. During the temperature optimization, the reaction was found a bit sluggish at below 60 °C; moreover, it took 6 h to produce 85% transformation at 80 °C (Table 1, entry 17). But, increasing the temperature, the duration of the reaction is drastically reduced, and at 110 °C, the reaction took just 15 min to brings the 90% transformation, while at 120, 130 and 150 °C, the reaction was completed in just 10, 7 and 2 min, respectively (Table 1, entries 18–20). In the present method, water was used as the solvent, and the reaction was refluxed between 100 and 110 °C. The solvent was also optimized, and the highest yield was achieved in water, which is a green and economical solvent.

Reaction also shows the significant transformations in methanol, ethanol, butanol, and *sec*-butyl alcohols however, isopropyl alcohol was not found suitable as it produces only 27% product (Table 1, entries 21–25). Other organic solvents, including toluene, 1,4-dioxane and dimethylformamide produce 45%, 19% and 5% yields of aniline, respectively (Table 1, entries 26–27). Besides hydrazine, other sources of hydrogen (Table 2), including isopropyl alcohol with KOH, and acetic acid or formic acid with triethylamine, were considered, but none of them mimicked the reaction. Moreover, not even a trace of product was observed when the reaction mixture was refluxed for up to 12 h. An insignificant transformation was obtained in an isopropyl alcohol and KOH solution, and it was not worth considering.

**Table 2:** Optimisation of reagents used as *in situ* hydrogen sources for the reaction^a^

S. no.	Reagents	% yield
1	Isopropyl alcohol and KOH	<20
2	Acetic acid with triethylamine	—
3	Formic acid with triethylamine	—
4	Hydrazine	89

a Nitrobenzene (1 mmol), catalyst Fe_3_Se_2_(CO)_9_ (3 mol%), temperature 110 °C, time 15 min, solvent water.

### Substrate scope

In order to check the scope of the reaction, various functionally different nitroarenes were screened at the optimized reaction parameters (Table 3). In general, most of the reactions produced excellent yield of the desired amine products. However, some variation was also observed with the change in the position of the functional group at the benzene ring. In comparison to *para*- and *meta*-substitution, the *ortho*-functionalised nitrobenzene derivatives show a bit less transformation. The electronic nature of the functional groups also influences the desired transformation, and a slightly better yield was obtained with electron-withdrawing group attached to nitrobenzene. The initial reaction of nitrobenzene produces an excellent 89% yield of reductive product, *i.e.*, aniline (1). Furthermore, the substrate scope was extended to halide derivatives of nitrobenzene: 4-chloro-, 4-bromo- and 4-iodonitrobenzene react smoothly and produce 86%, 84% and 83% yield of the respective aniline products. The position of the substituent also influences the productivity: a slightly reduced yield was obtained with 2-bromonitro benzene (5, 79%). 2,5-di-bromonitrobenzene also reacted efficiently and produced an excellent yield (6, 82%) of the respective product. Various electron-withdrawing groups were also tested for the present reaction, *para*-hydroxy and *para*-cyanonitrobenzene both yield highly significant transformations (7, 87%) and (8, 85%) of the respective aniline products. The 1,4-dinitrobenzene reacted under the optimised conditions to give an outstanding yield with a selective reduction of one nitro group, and *para*-nitroaniline (9) was isolated with 88% transformation. Moreover, a 75% transformation of 1,4-diaminobenzene (10) was observed when the same reaction was repeated with an increased quantity of hydrazine. A slightly reduced reduction of 2-aminonitrobenzene to 1,2-diaminobenzene (11, 76%) was observed due to the *ortho* positions. However, a significantly enhanced yield (12, 86%) of the *meta*-substituted aminonitrobenzene was recorded. A similar reduced trend was also observed with other *ortho*- and *para*-functional derivatives. The *ortho*- and *para*-derivatives of methyl nitrobenzene produced 71% (13) and 73% (14) transformation of their respective products. A marginally increased yield was recorded with *para*-methoxy nitrobenzene (15, 80%). Functional group interference was expected, but *para*-nitro acetophenone produced an excellent 83% (16) yield of desired product. Furthermore, the reaction of *para*-nitro benzyl alcohol conveniently react to produce 79% (17) of the corresponding product. Similarly, 1-nitronaphthalene also participated well in the reaction to produce 87% (18).

**Table 3:** General scope of the reaction with various derivatives of nitrobenzene^a^

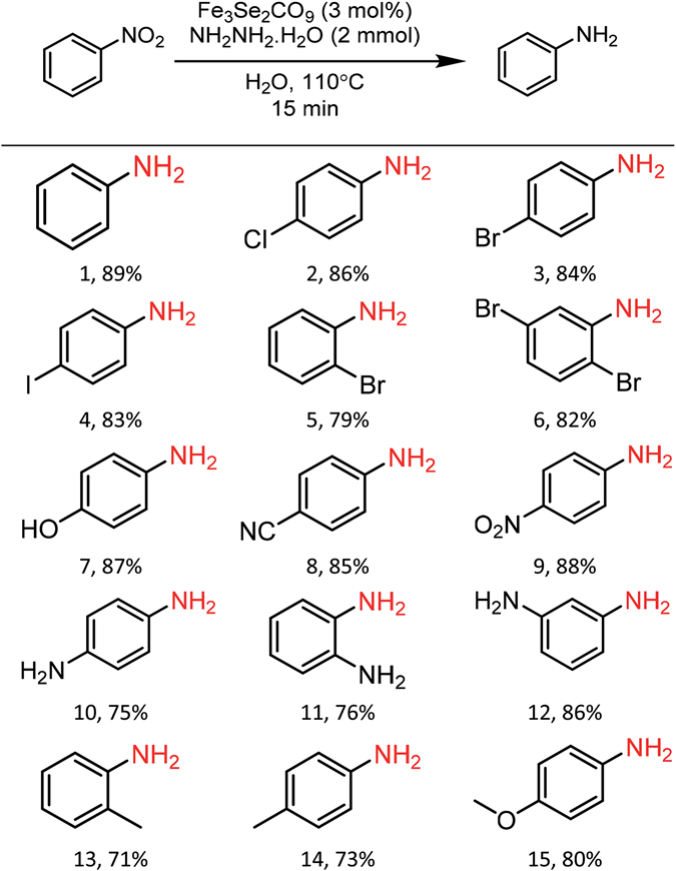

a Reaction conditions: Fe_3_Se_2_(CO)_9_ (3 mol%), nitrobenzene derivatives (1 mmol), N_2_H_4_·H_2_O (2 mmol), temperature 110 °C, time 15 min, solvent water, isolated yields.

### Catalyst recycling

The catalyst was isolated from the organic layer and used again with an aqueous layer for another catalytic cycle. In the second and third catalytic cycles, 63 and 46% transformations of the desired product were obtained, respectively. This indicates the decreasing catalytic efficiency of the catalyst. Besides this, it was also noted that there was 20% loss of catalyst in each catalytic run. This may be due to the presence of moisture on the surface of the catalyst, as a broad signal for moisture (at 3409 cm^−1^) was recorded in the FTIR spectrum of the used catalyst (Fig. S1, ESI†). Therefore, the catalyst has to be dried in an oven for 1 h before using it for the next catalytic cycle.

### Plausible mechanism

**Scheme 3:**
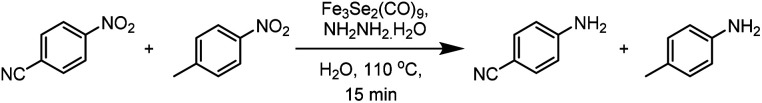
Control experiment showing affinity towards the withdrawing group.

While performing control experiments it was observed that the reaction has good selectivity towards the electron-withdrawing functionalities. A reaction mixture of 4-methylnitrobenzene and 4-cyanonitrobenzene (1 equivalent of each) under optimised reaction condition produces *para*-cyano aniline (73%) as a major product and *para*-methyl aniline (27%) as minor product (Scheme 3).

In another control experiment maintaining similar reaction conditions and the same reagents, after 15 minutes of the reaction, furthermore 2 equiv. of hydrazine was added and the reaction was again run for next 15 minutes. After two consecutive cycles, 91 and 56% yields of *p*-cyano aniline and *p*-methyl aniline were obtained respectively. In the second run, the required hydrazine was added, but the activity of the catalyst was considerably compromised. Therefore only a marginal increase in the yield of *p*-cyano aniline was observed, while due to the significant quantity of unreacted 4-methyl nitrobenzene it showed a significant increase in transformations.

**Scheme 4:**
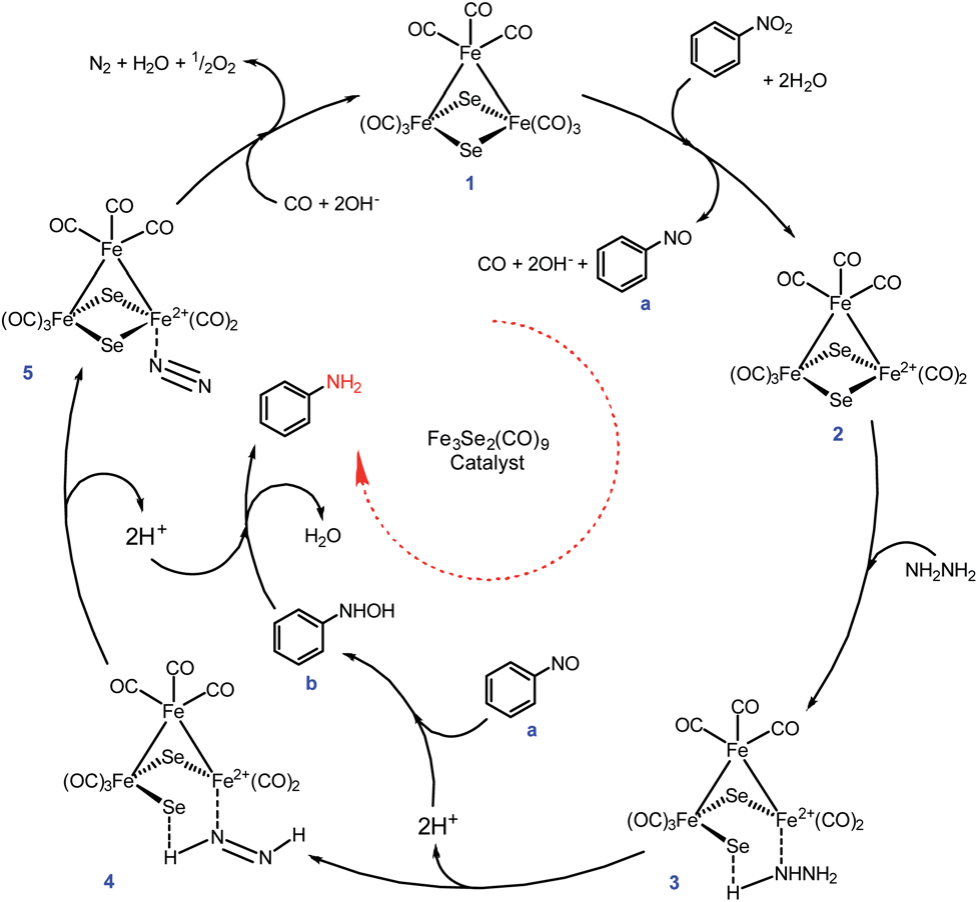
A plausible mechanism for the nitro reduction.

However, neither a detailed study conducted nor any intermediate species were isolated during the present transformation, but a tentative mechanism has been proposed on the basis of evidence available in the literature (Scheme 4). There are strong evidences in the literature suggests that Fe(0) and Se both are individually excellent catalysts for nitro reduction.^48–55^

Initially, the Fe-cluster (1) interacts with nitrobenzene in the presence of water and is oxidised and releases the required electrons with the simultaneous loss of CO to form intermediate (2), which facilitates the formation of nitrosobenzene (a).^53^ The hydrazine coordinates with intermediate (2) to produce (3). The interaction of the proton present in the close vicinity of the bridged Se in intermediate (3) leads to the loss of the Fe–Se bond. The deprotonation of intermediate (3) results in intermediate (4). Moreover, a simultaneous protonation of nitrosobenzene forms intermediate arylhydroxylamine (b). Further consecutive deprotonation of intermediate 4 and protonation of arylhydroxylamine form intermediate 5 and arylamine, respectively. Loss of N_2_ and reunion of CO with intermediate 5 regenerate the catalyst (1). The nitro reduction is possible *via* either four or six electron transfer. The six electron transfer strongly needs an alkaline medium^47^ and during the six electron transfer, nitrosobenzenes may undergo condensation. This is quite rare, but the formation of azoxybenzene followed by azobenzene and hydrazobenzene to aniline is frequently observed.^50^

## Experimental section

### General procedure for the catalytic synthesis

In a clean reaction tube were placed the Fe_3_Se_2_(CO)_9_ catalyst (13.5 mg, 3 mol%) and the derivative of nitroarenes (1 mmol). To this were added hydrazine hydrate (64 μL, 2 mmol) and water as a solvent, the reaction tube was heated with continuous stirring at 110 °C for 15 min. After completion of the reaction, the reaction mixture was cooled to room temperature, and the desired organic product was extracted in an organic layer of ethyl acetate. The organic layer was then dried over anhydrous Na_2_SO_4_, and ethyl acetate was evaporated on a rotavapor at reduced pressure to obtain the crude product. Finally, the crude product was subjected to a chromatographic work-up and further purification and spectral characterization.

## Conclusion

In conclusion, a robustly stable, reusable, highly economical, easy to synthesise and zero-valent iron chalcogenide carbonyl cluster Fe_3_Se_2_(CO)_9_ has been explored for the reduction of nitroarenes. The chalcogen-stabilised Fe-cluster brings about an excellent transformation to a range of functionalised nitroarenes under the inert free conditions and in an aqueous medium. The methodology was found to be strongly feasible and worked in the green solvent water. Moreover, it also avoids the use of a precious metal catalyst or supporting reagents. The catalyst can be reused for three catalytic cycles. The present methodology also provides a selective but temperature-dependent timeframe (2–15 min) to accomplish the reaction. This is the first report where zero-valent chalcogen-stabilised iron carbonyl clusters were explored for nitro reduction in the absence of high CO pressure.

## Conflicts of interest

There are no conflicts to declare.

## Supplementary Material

RA-010-D0RA04491A-s001
