# Supplementary material for: CO-free, aqueous mediated, instant and selective reduction of nitrobenzene via robustly stable chalcogen stabilised iron carbonyl clusters (Fe3E2(CO)9, E = S, Se, Te)
Source: RSC Adv. 2020 Sep 1;10(54):32516–21. doi: 10.1039/d0ra04491a (PMC9056603; doi:10.1039/d0ra04491a)
Supplement: RA-010-D0RA04491A-s001 [file RA-010-D0RA04491A-s001.pdf]

Electronic Supplementary Material (ESI) for RSC Advances.
This journal is © The Royal Society of Chemistry 2020

SUPPORTING INFORMATION

CO-free, Aqueous Mediated, Instant and Selective Reduction of Nitrobenzene via Robustly Stable Chalcogen Stabilised Iron carbonyl Clusters ($Fe_3E_2(CO)_9$, E= S, Se, Te)

Charu Sharma, Avinash K. Srivastava, Aditi Soni, Sangeeta Kumari, Raj K. Joshi*

Department of Chemistry, Malaviya National Institute of Technology, Jaipur 302017, Rajasthan, India, Email: rkjoshi.chy@mnit.ac.in

Experimental Details: The ^1H, ^{13}C {^1H} NMR spectra were recorded using JEOL ECS-400 spectrometer (operating at 400 MHz for ^1H and 100 MHz for ^{13}C).

Chemicals and reagents: Reactants, reagents, chemicals and solvents available commercially within the country were used.

Experimental Section

In a clean reaction tube, take $Fe_3Se_2(CO)_9$ catalyst (3 mol%) and derivative of nitroarenes (1 mmol). To this added hydrazine hydrate (2 mmol) and water as a reaction medium. Above mixture is heated at 110°C for 15 min. Through TLC monitoring product formation was investigated. After that reaction mixture was cooled at room temperature, by adding water and EtOAc the organic layer was extracted. By using anhydrous Na_2SO_4 extracted layer was dried. Under reduced pressure solvent was evaporated to get the crude product. Finally, the product was purified through column chromatography.

Characterisation Data

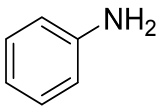	**Aniline**[1]<br>**1H NMR (400MHz, CDCl3)**: δ = 7.23-7.18 (m, 2H), 6.82-6.79 (t, 1H), 6.72-6.70 (t, 2H), 3.63 (s, 2H)<br>**13C NMR (100MHz, CDCl3)**: δ = 146.33, 129.42, 118.70, 115.03
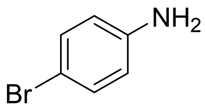	**4-Bromoaniline**[2]<br>**1H NMR (400MHz, CDCl3)**: δ = 7.11-7.07 (m, 2H), 6.51-6.48 (m, 2H), 5.21 (s, 2H)<br>**13C NMR (100MHz, CDCl3)**: δ = 148.52, 131.85, 116.34, 106.68
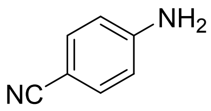	**4-Aminobenzonitrile**[3]<br>**1H NMR (400MHz, CDCl3)**: δ = 7.38-7.34 (m, 2H), 6.63-6.60 (m, 2H), 4.25 (s, 2H)<br>**13C NMR (100MHz, CDCl3)**: δ = 150.76, 133.77, 113.97, 99.95
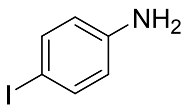	**4-Iodoaniline**[4]<br>**1H NMR (400MHz, CDCl3)**: δ = 7.41-7.37 (m, 2H), 6.51-6.44 (m, 2H), 3.53 (s, 2H)<br>**13C NMR (100MHz, CDCl3)**: δ = 145.56, 137.83, 117.25, 78.87
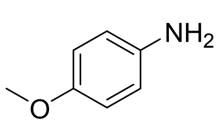	**4-methoxyaniline**[5]<br>**1H NMR (400MHz, CDCl3)**: δ = 6.76-6.72 (m, 2H), 6.65-6.61 (m, 2H), 3.73 (s, 3H), 3.42 (s, 2H)<br>**13C NMR (100MHz, CDCl3)**: δ = 152.88, 140.12, 116.51, 114.92, 55.33
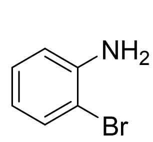	**2-Bromoaniline**[6]<br>**1H NMR (400MHz, CDCl3)**: δ = 7.29-7.27 (m, 1H), 7.03-6.98 (m, 1H), 6.76-6.73 (m, 1H), 6.44-6.40 (m, 1H), 5.23 (s, 2H)<br>**13C NMR (100MHz, CDCl3)**: δ = 146.28, 132.62, 128.80, 117.85, 115.92, 107.95

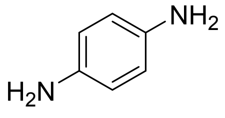	**Benzene-1,4-diamine**[7]<br>**1H NMR (400MHz, CDCl3)**: δ = 6.55 (s, 4H), 3.28 (s, 4H)<br>**13C NMR (100MHz, CDCl3)**: δ = 138.85, 116.42
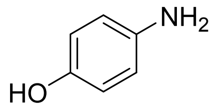	**4-Aminophenol**[8]<br>**1H NMR (400MHz, CDCl3)**: δ = 8.30 (s, 1H), 6.44-6.36 (m, 4H), 4.35 (s, 2H)<br>**13C NMR (100MHz, CDCl3)**: δ =148.73, 141.16, 116.04, 115.74
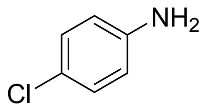	**4-Chloroaniline**[1]<br>**1H NMR (400MHz, CDCl3)**: δ = 7.10-7.08 (m, 2H), 6.60-6.57 (m, 2H), 3.65 (s, 2H)<br>**13C NMR (100MHz, CDCl3)**: δ = 144.50, 129.42, 123.16, 115.80
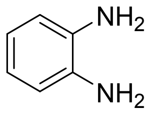	**Benzene-1,2-diamine**[7]<br>**1H NMR (400MHz, CDCl3)**: δ = 6.73-6.68 (m, 4H), 3.32 (s, 4H)<br>**13C NMR (100MHz, CDCl3)**: δ = 134.82, 120.37, 116.84
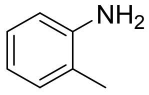	**o-Toluidine**[7]<br>**1H NMR (400MHz, CDCl3)**: δ = 7.05-7.01 (t, 2H), 6.72-6.66 (m, 2H), 3.59 (s, 1H), 2.14 (s, 3H)<br>**13C NMR (100MHz, CDCl3)**: δ = 144.12, 130.88, 126.82, 121.69, 119.09, 113.97, 18.09
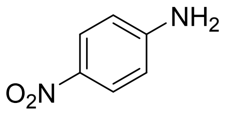	**4-Nitroaniline**[7]<br>**1H NMR (400MHz, CDCl3)**: δ = 8.07-8.05 (d, 2H), 6.63-6.59(m, 2H), 4.35 (s, 4H)<br>**13C NMR (100MHz, CDCl3)**: δ = 152.11, 139.15, 126.16, 113.18
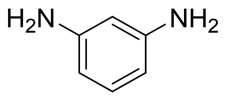	**Benzene-1,3-diamine**[7]<br>**1H NMR (400MHz, CDCl3)**: δ = 6.95-6.91 (t, 1H), 6.12-6.10 (m, 2H), 6.02-6.01 (d, 1H)<br>**13C NMR (100MHz, CDCl3)**: δ = 147.61, 130.29, 105.55, 102.27

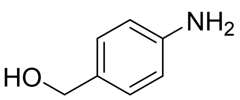	**(4-Aminophenyl)methanol**[4]<br>**1H NMR (400MHz, CDCl3)**: δ = 7.16-7.13 (d, 2H), 6.67-6.64 (m, 4H), 4.53 (s, 2H)<br>**13C NMR (100MHz, CDCl3)**: δ = 146.15, 131.49, 129.11, 114.66, 65.42
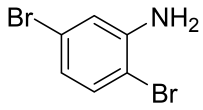	**2,5-Dibromoaniline**[2]<br>**1H NMR (400MHz, CDCl3)**: δ = 7.24-7.22 (d, 1H), 6.91-6.88 (t, 1H), 6.73- 6.67 (m, 1H), 4.12 (s, 2H)<br>**13C NMR (100MHz, CDCl3)**: δ = 145.96, 144.49, 133.10, 121.98, 117.25, 108.07
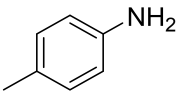	**p-Toluidine**[4]<br>**1H NMR (400MHz, CDCl3)**: δ = 6.99-6.97 (d, 2H), 6.63-6.61 (d, 2H), 3.53 (s, 2H), 2.26 (s, 3H)<br>**13C NMR (100MHz, CDCl3)**: δ = 143.94, 129.42, 127.66, 115.37, 20.33
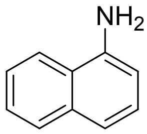	**naphthalen-1-amine**[2]<br>**1H NMR (400MHz, CDCl3)**: δ = 7.86-7.80 (m, 2H), 7.51- 7.46 (m, 2H), 7.37-7.31 (m, 2H), 6.80-6.78 (d, 1H), 4.41 (s, 2H)<br>**13C NMR (100MHz, CDCl3)**: δ = 142.21, 134.51, 128.69, 126.56, 125.95, 125.00, 123.69, 120.96, 119.07, 109.73
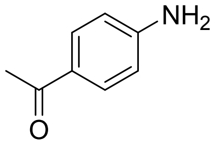	**1-(3-aminophenyl)ethan-1-one**<br>**1H NMR (400MHz, CDCl3)**: δ = 7.31-7.29 (d, 2H), 7.24-7.19 (m, 2H), 6.86-6.84 (m, 1H), 3.82 (s, 2H), 2.54 (s, 3H)<br>**13C NMR (100MHz, CDCl3)**: δ = 198.81, 146.73, 137.84, 129.43, 119.47, 114.26, 99.56, 26.60

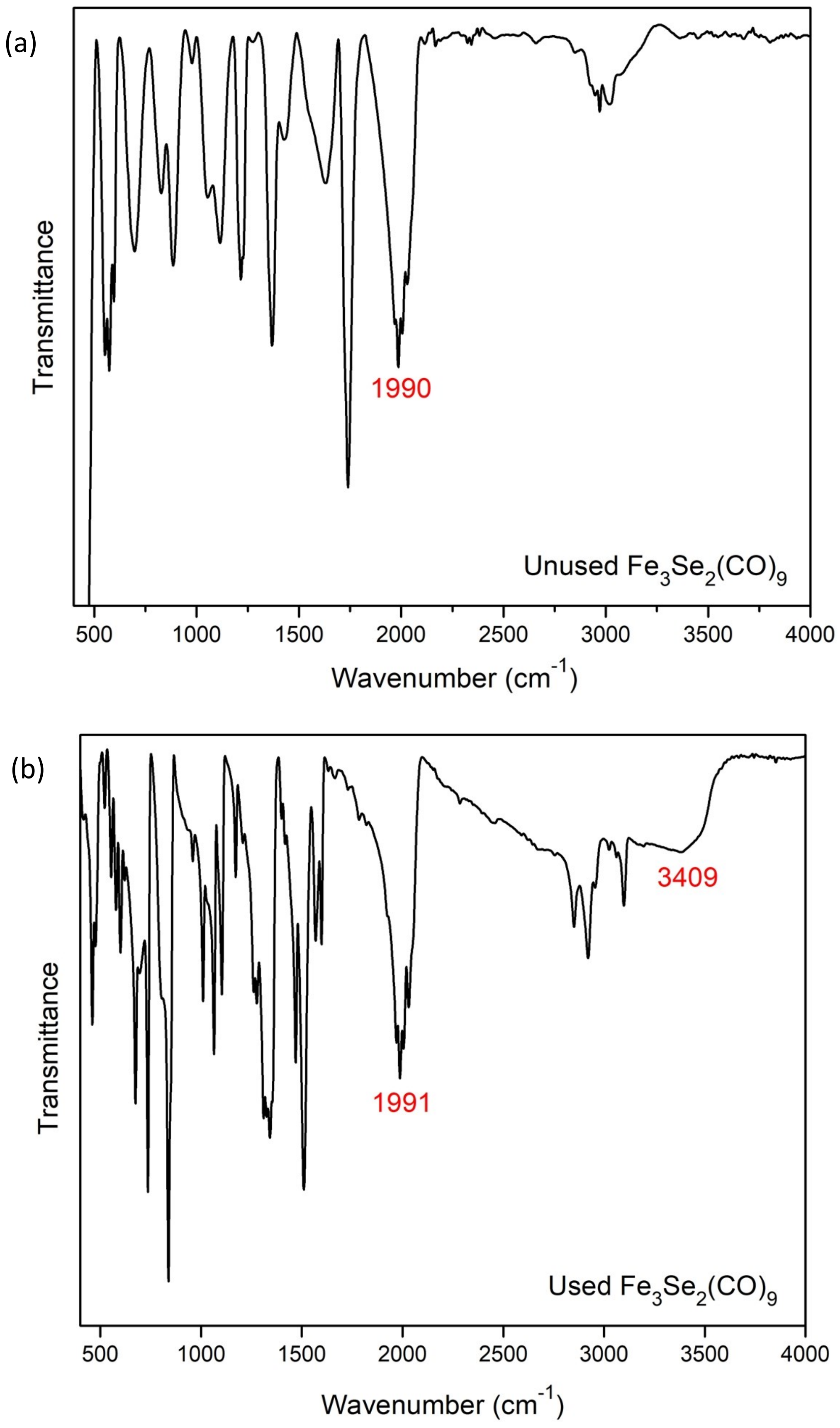

Figure s1: (a) FTIR spectrum of Unused $Fe_3Se_2(CO)_9$, (b) FTIR spectrum of Used $Fe_3Se_2(CO)_9$

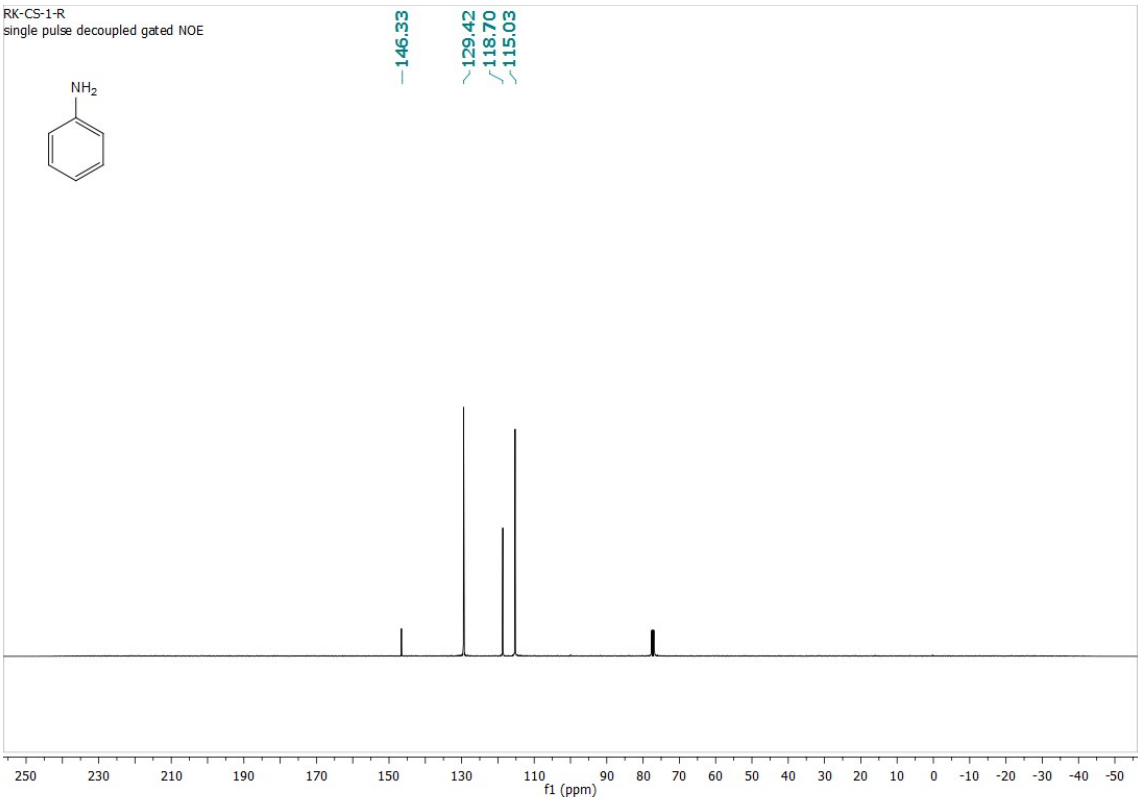
RK-CS-1-R
single pulse decoupled gated NOE
NH2
146.33
129.42
118.70
115.03
f1 (ppm)

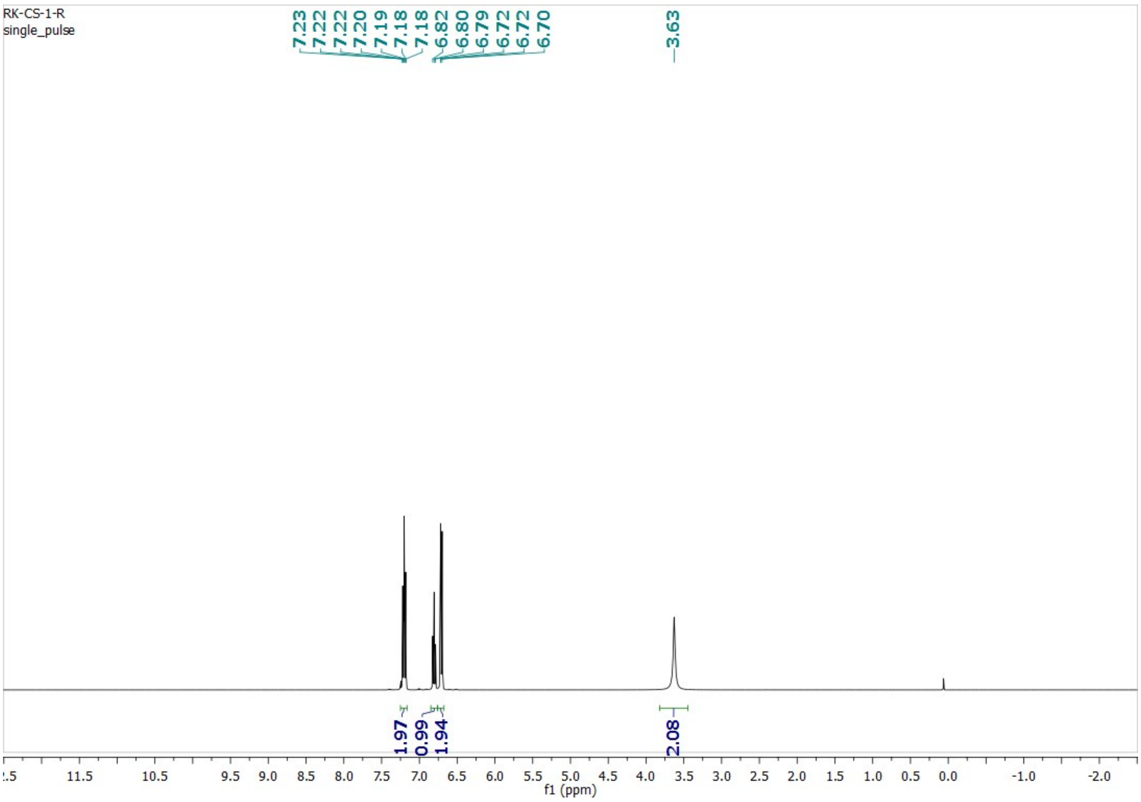
RK-CS-1-R
single_pulse
7.23
7.22
7.22
7.20
7.19
7.18
7.18
6.82
6.80
6.79
6.72
6.72
6.70
3.63
1.97
0.99
1.94
2.08
f1 (ppm)

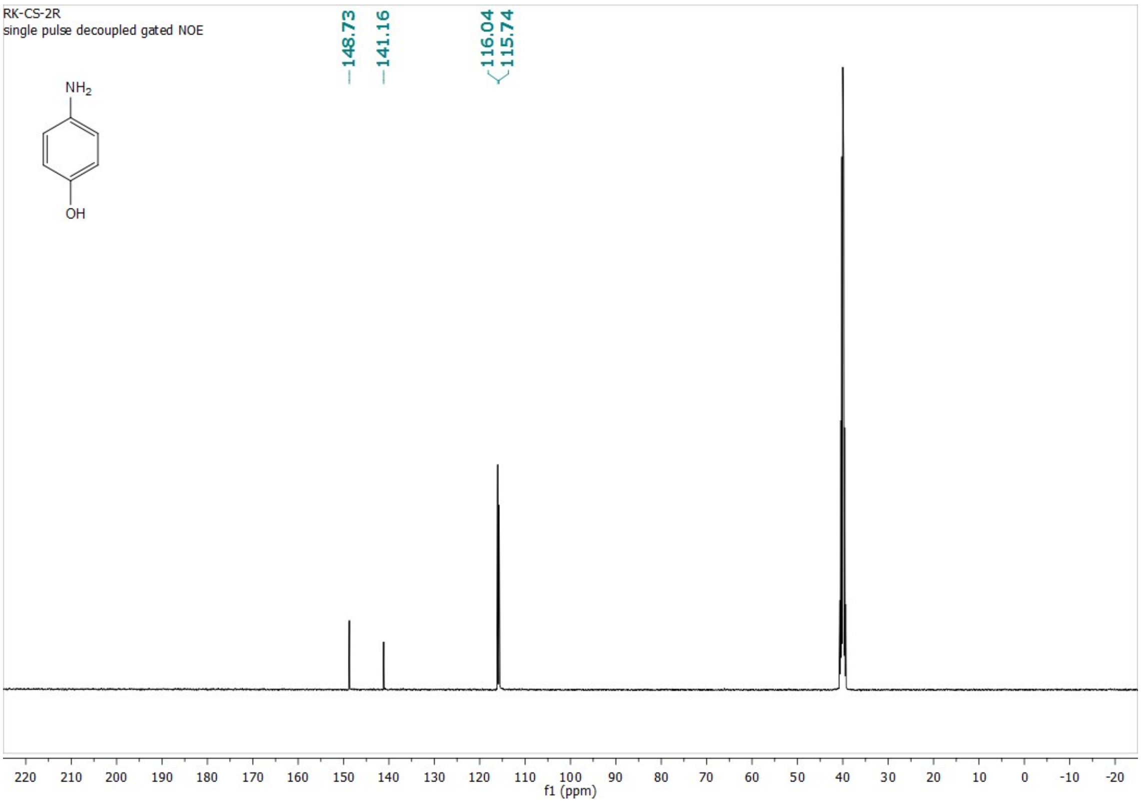
RK-CS-2R
single pulse decoupled gated NOE
NH2
OH
148.73
141.16
116.04
115.74
220 210 200 190 180 170 160 150 140 130 120 110 100 90 80 70 60 50 40 30 20 10 0 -10 -20
f1 (ppm)

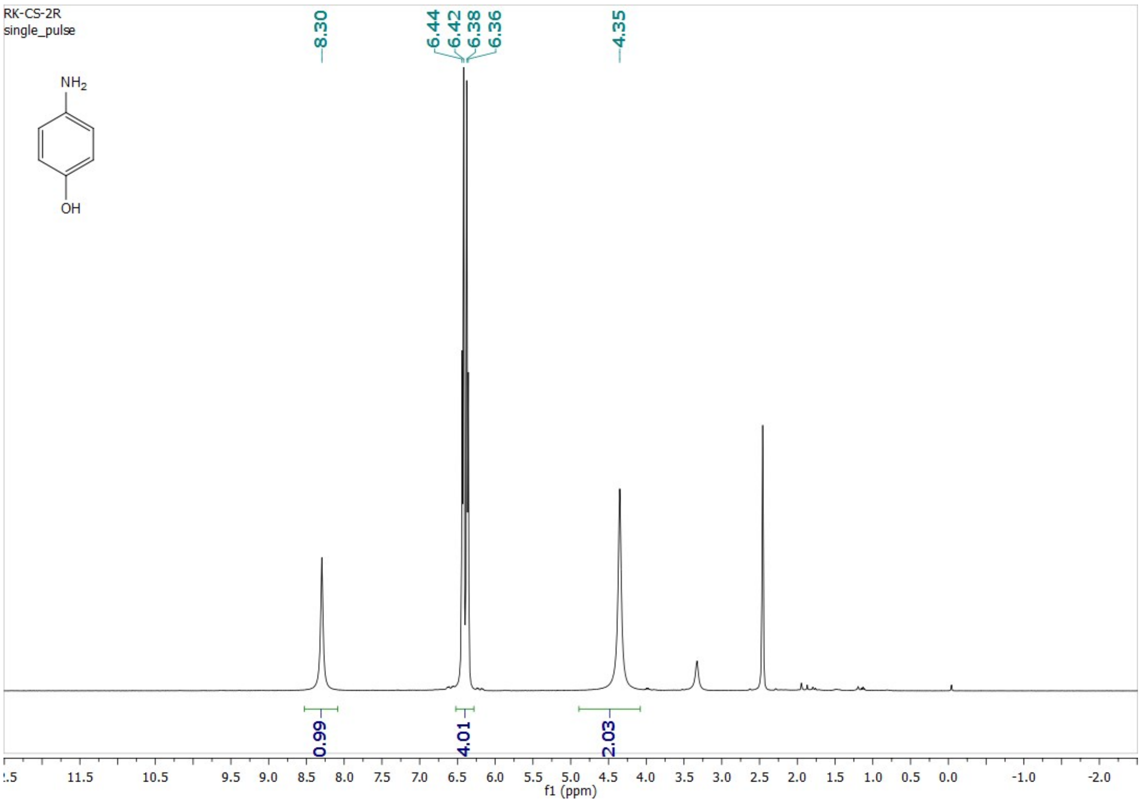
RK-CS-2R
single_pulse
NH2
OH
8.30
6.44
6.42
6.38
6.36
4.35
0.99
4.01
2.03
11.5 10.5 9.5 9.0 8.5 8.0 7.5 7.0 6.5 6.0 5.5 5.0 4.5 4.0 3.5 3.0 2.5 2.0 1.5 1.0 0.5 0.0 -1.0 -2.0
f1 (ppm)

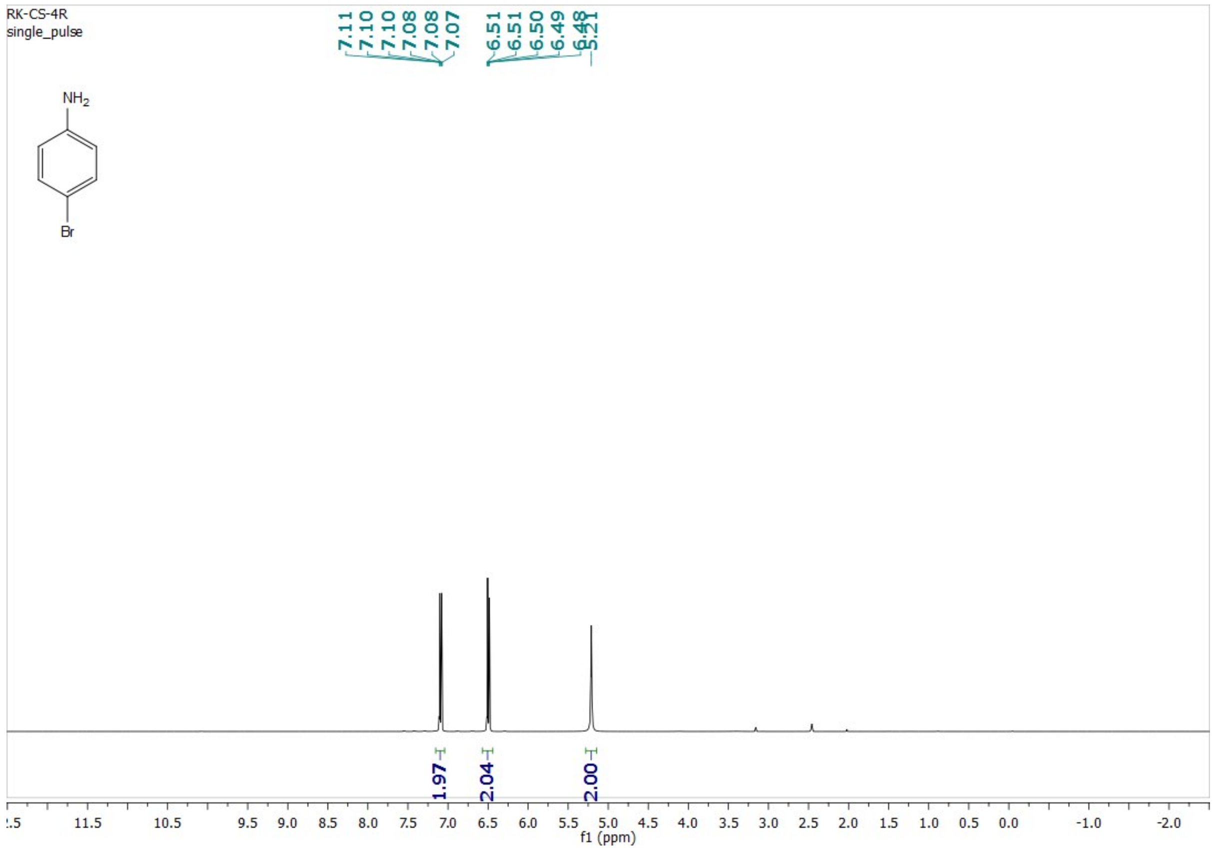
RK-CS-4R
single_pulse
NH2
Br
7.11
7.10
7.10
7.08
7.08
7.07
6.51
6.51
6.50
6.49
6.48
5.21
1.97
2.04
2.00
f1 (ppm)

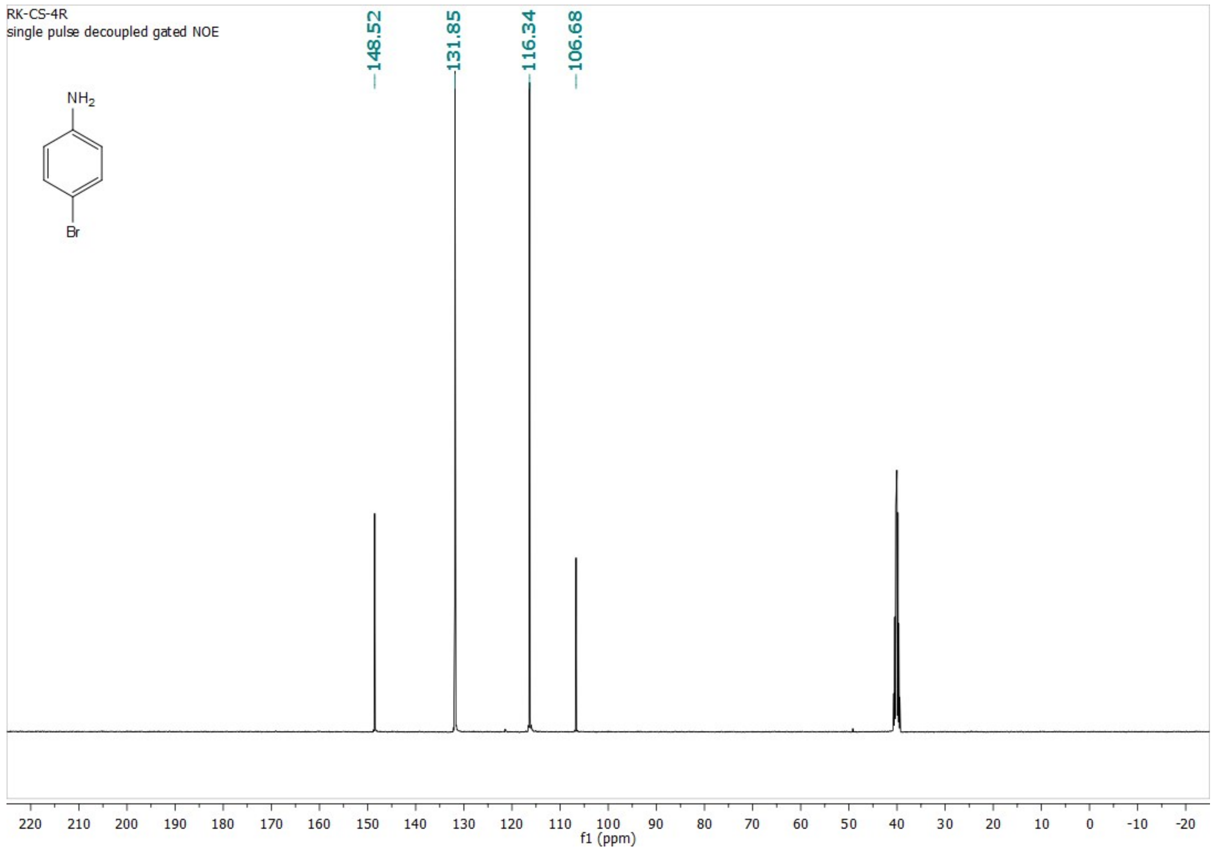
RK-CS-4R
single pulse decoupled gated NOE
NH2
Br
148.52
131.85
116.34
106.68
f1 (ppm)

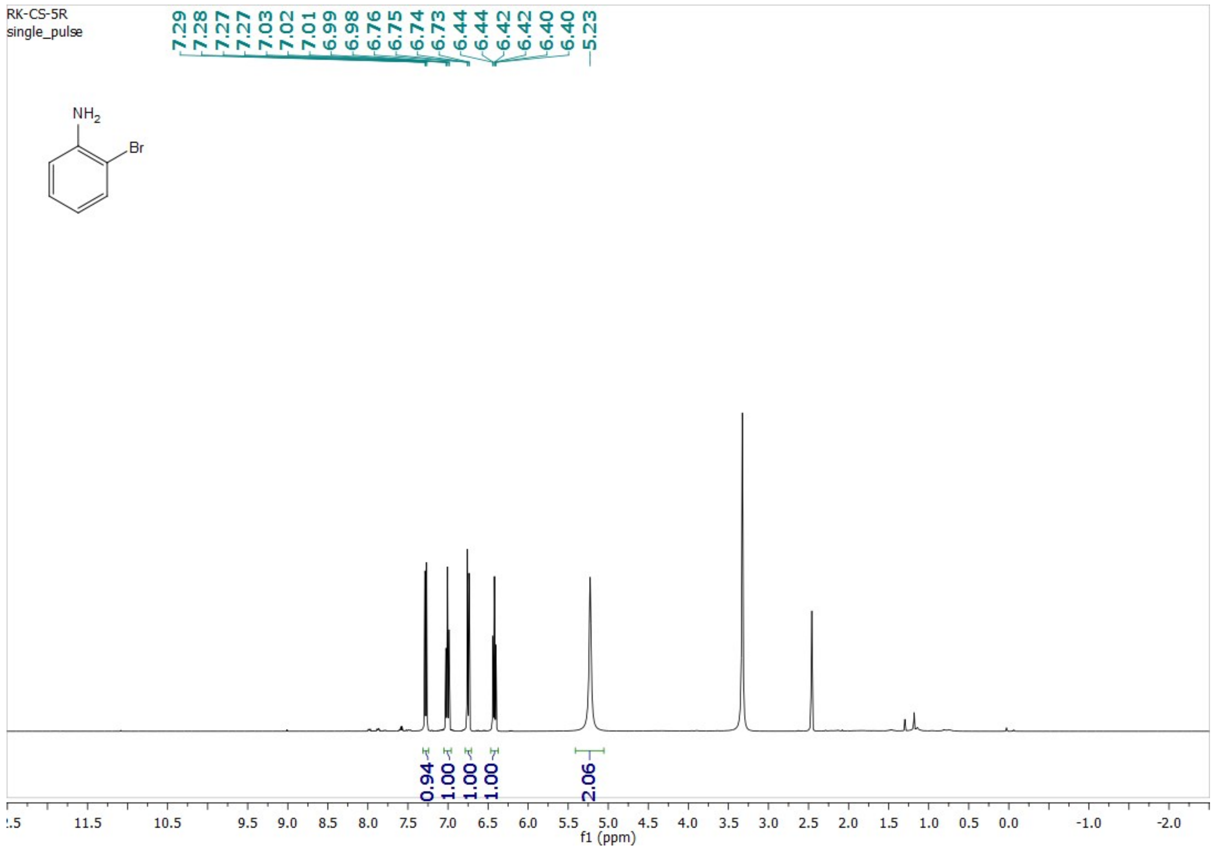
RK-CS-5R
single_pulse
7.29
7.28
7.27
7.27
7.03
7.02
7.01
6.99
6.98
6.76
6.75
6.74
6.73
6.44
6.44
6.42
6.42
6.40
6.40
5.23
NH2
Br
0.94
1.00
1.00
1.00
2.06
f1 (ppm)

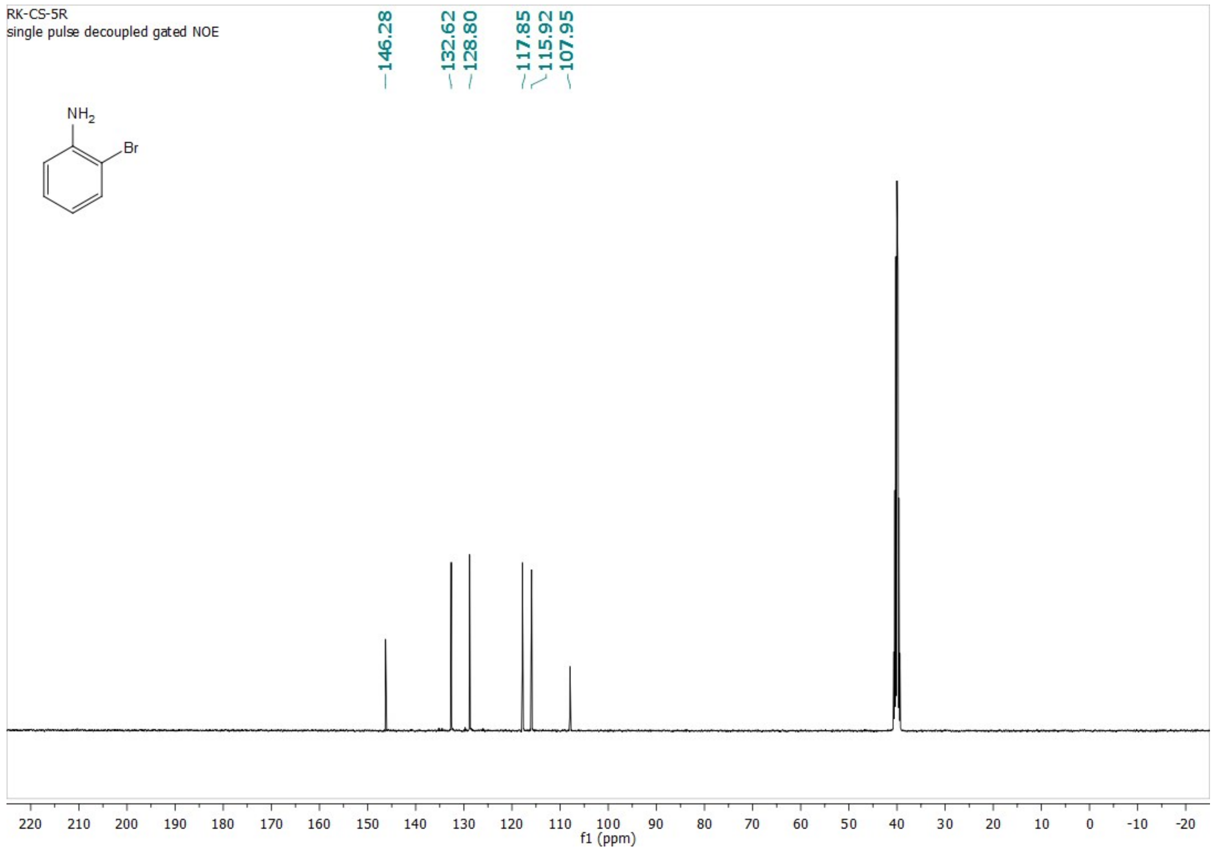
RK-CS-5R
single pulse decoupled gated NOE
146.28
132.62
128.80
117.85
115.92
107.95
NH2
Br
f1 (ppm)

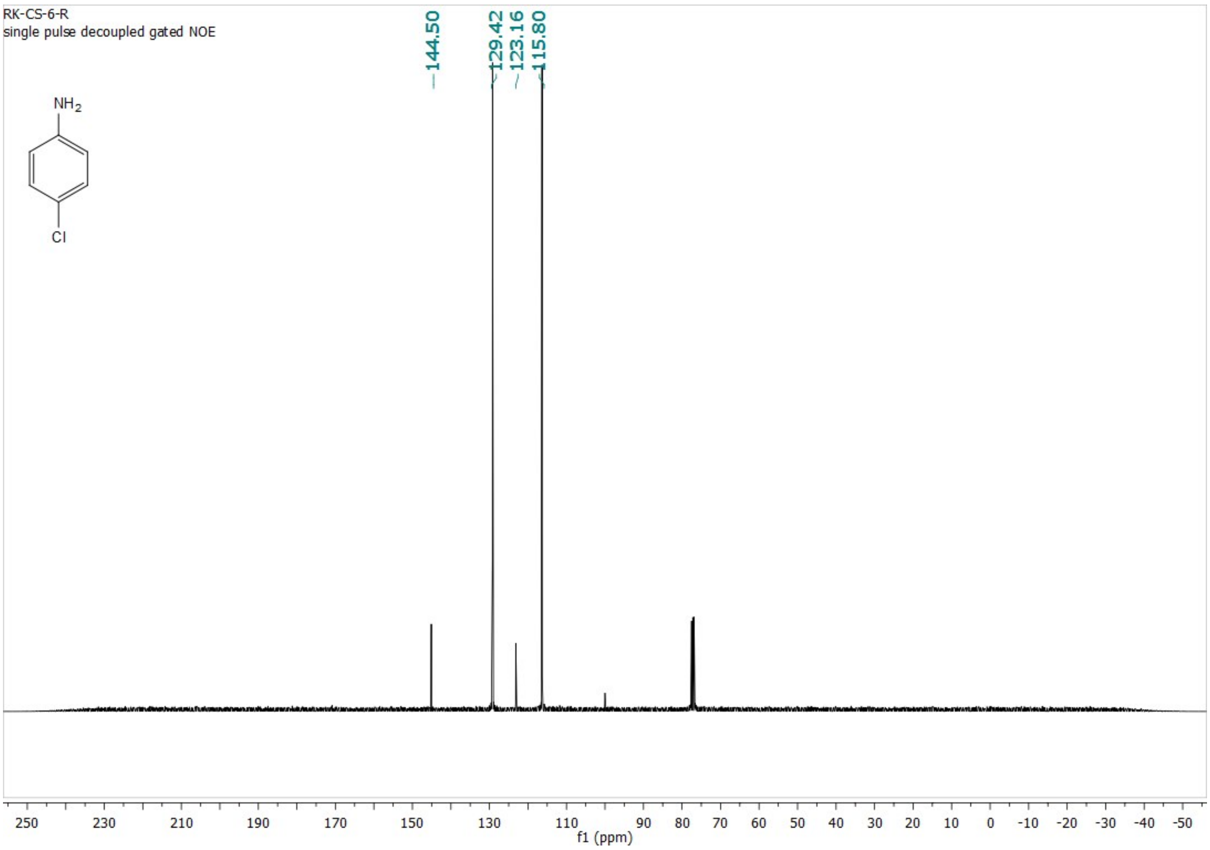
RK-CS-6-R
single pulse decoupled gated NOE
NH2
Cl
144.50
129.42
123.16
115.80
250 230 210 190 170 150 130 110 90 80 70 60 50 40 30 20 10 0 -10 -20 -30 -40 -50
f1 (ppm)

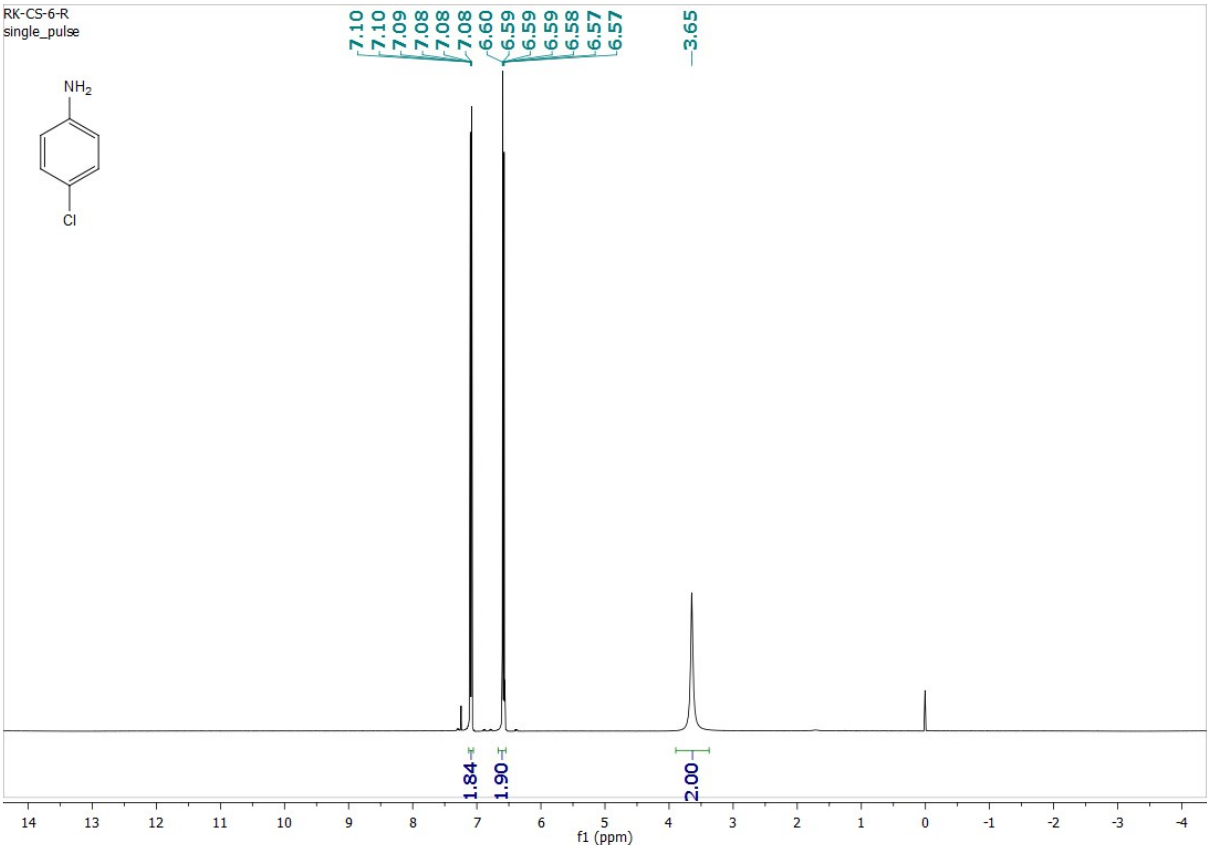
RK-CS-6-R
single_pulse
NH2
Cl
7.10
7.10
7.09
7.08
7.08
7.08
6.60
6.59
6.59
6.59
6.58
6.57
6.57
3.65
1.84
1.90
2.00
14 13 12 11 10 9 8 7 6 5 4 3 2 1 0 -1 -2 -3 -4
f1 (ppm)

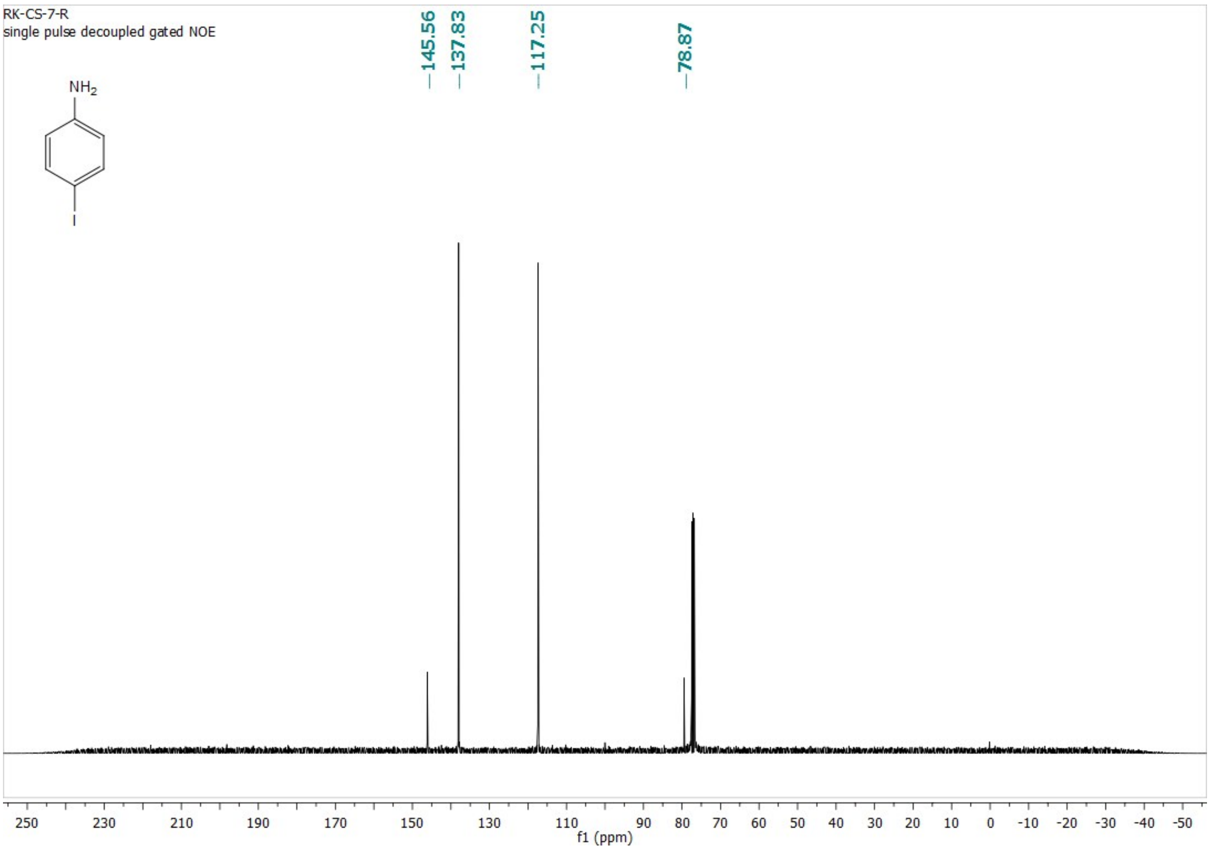
RK-CS-7-R
single pulse decoupled gated NOE
NH2
I
145.56
137.83
117.25
78.87
250 230 210 190 170 150 130 110 90 80 70 60 50 40 30 20 10 0 -10 -20 -30 -40 -50
f1 (ppm)

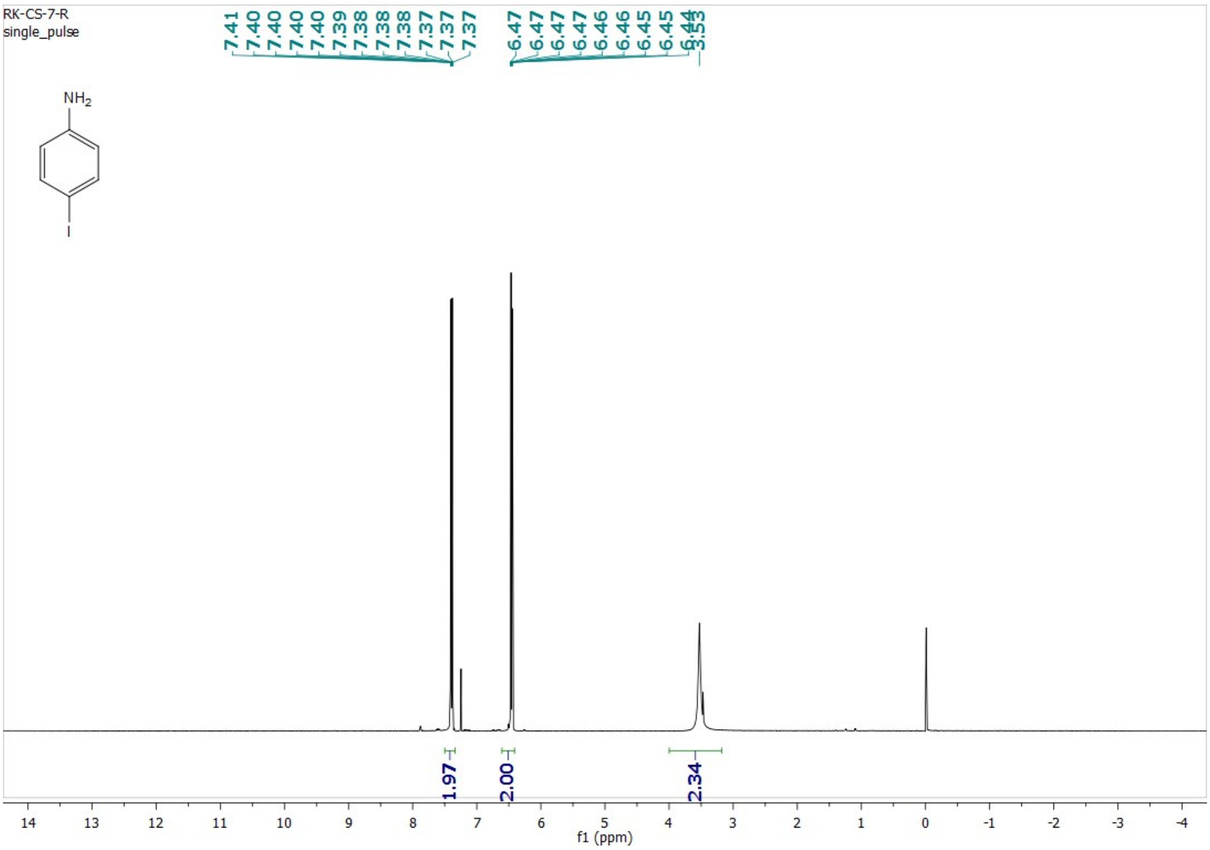
RK-CS-7-R
single_pulse
NH2
I
7.41 7.40 7.40 7.40 7.40 7.39 7.38 7.38 7.38 7.37 7.37 7.37
6.47 6.47 6.47 6.47 6.46 6.46 6.45 6.45 6.44
3.53
1.97
2.00
2.34
14 13 12 11 10 9 8 7 6 5 4 3 2 1 0 -1 -2 -3 -4
f1 (ppm)

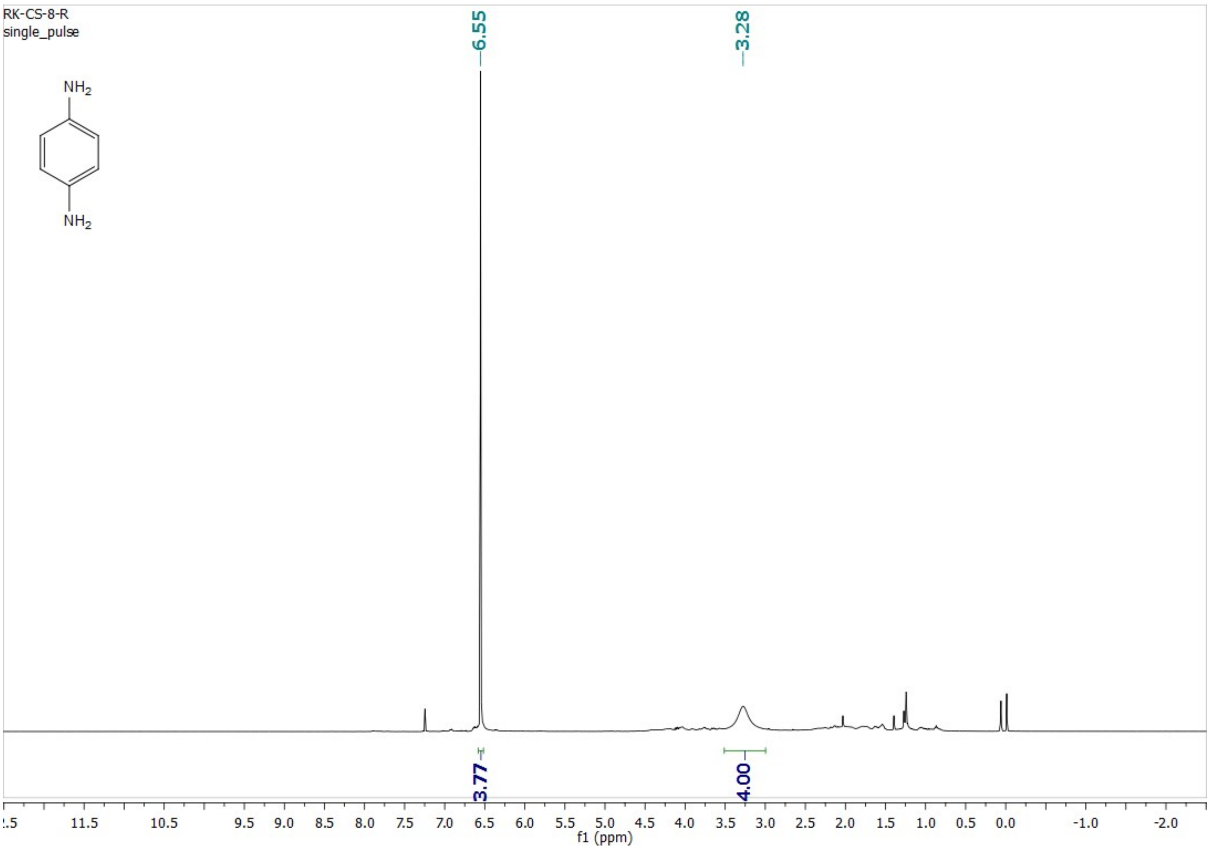
RK-CS-8-R
single_pulse
NH2
NH2
6.55
3.28
3.77
4.00
f1 (ppm)

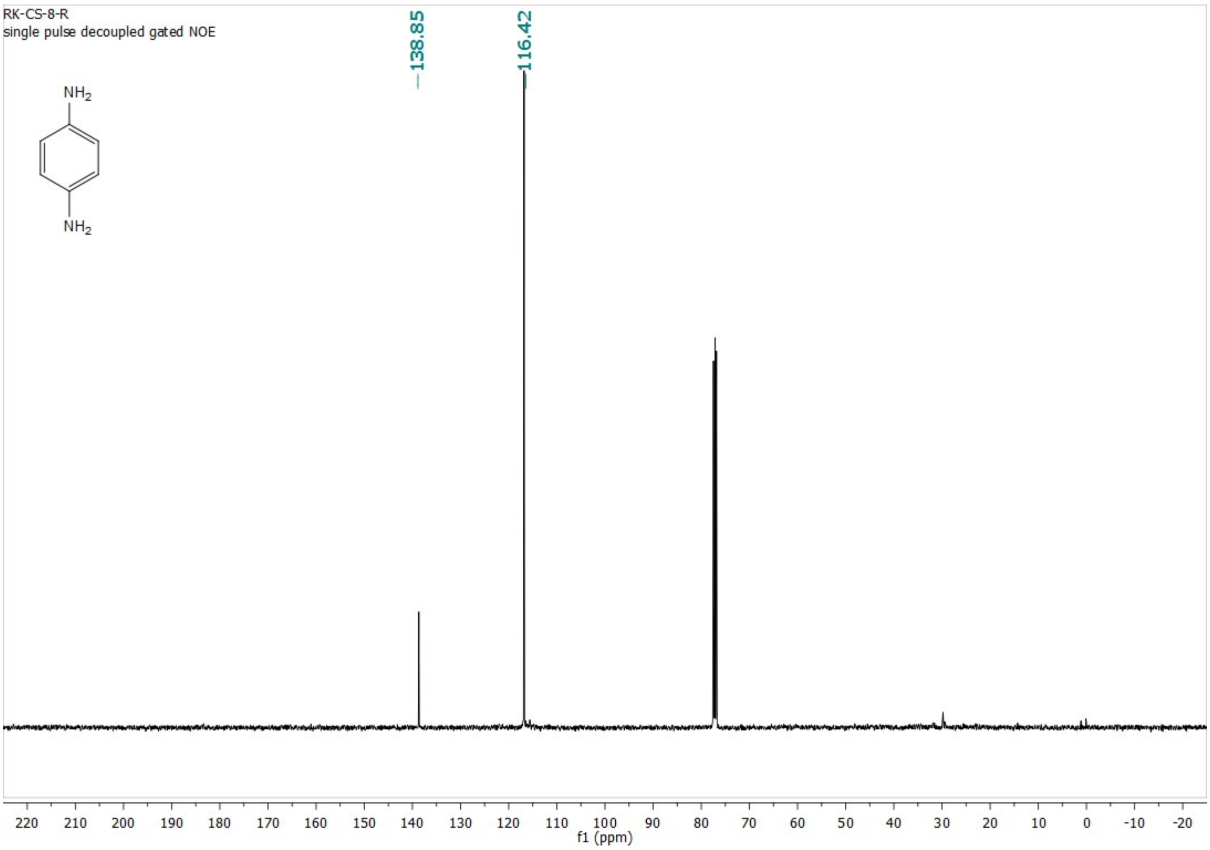
RK-CS-8-R
single pulse decoupled gated NOE
NH2
NH2
138.85
116.42
f1 (ppm)

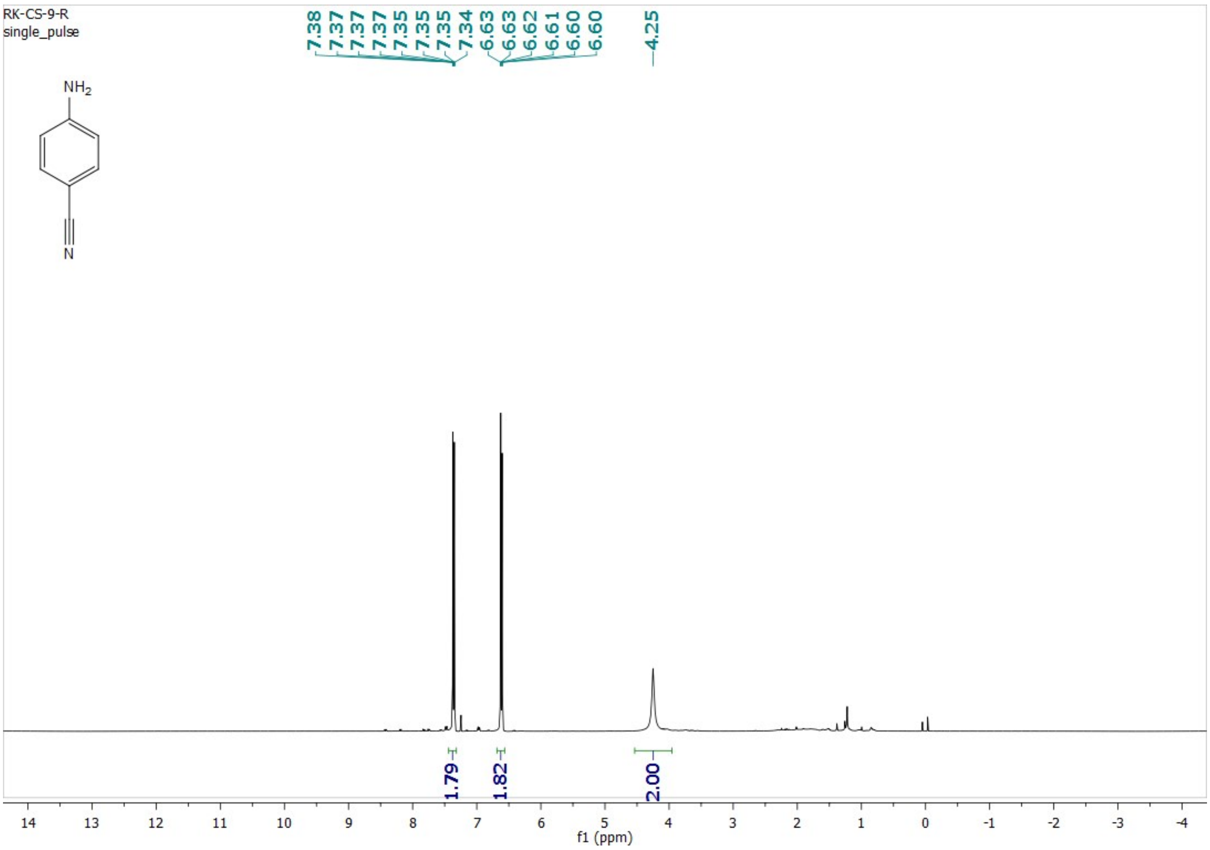
RK-CS-9-R
single_pulse
NH2
N
7.38
7.37
7.37
7.37
7.35
7.35
7.35
7.34
6.63
6.63
6.62
6.61
6.60
6.60
4.25
1.79
1.82
2.00
14 13 12 11 10 9 8 7 6 5 4 3 2 1 0 -1 -2 -3 -4
f1 (ppm)

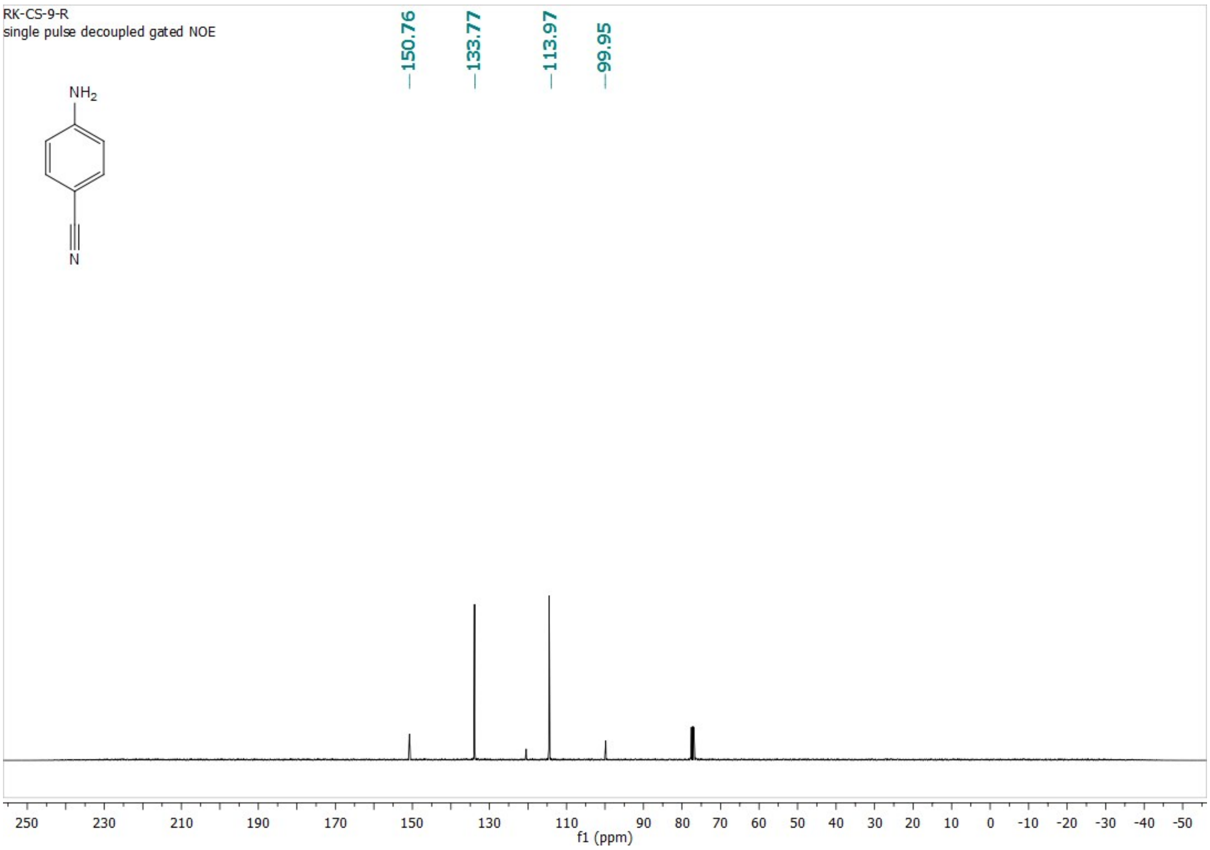
RK-CS-9-R
single pulse decoupled gated NOE
NH2
N
150.76
133.77
113.97
99.95
250 230 210 190 170 150 130 110 90 80 70 60 50 40 30 20 10 0 -10 -20 -30 -40 -50
f1 (ppm)

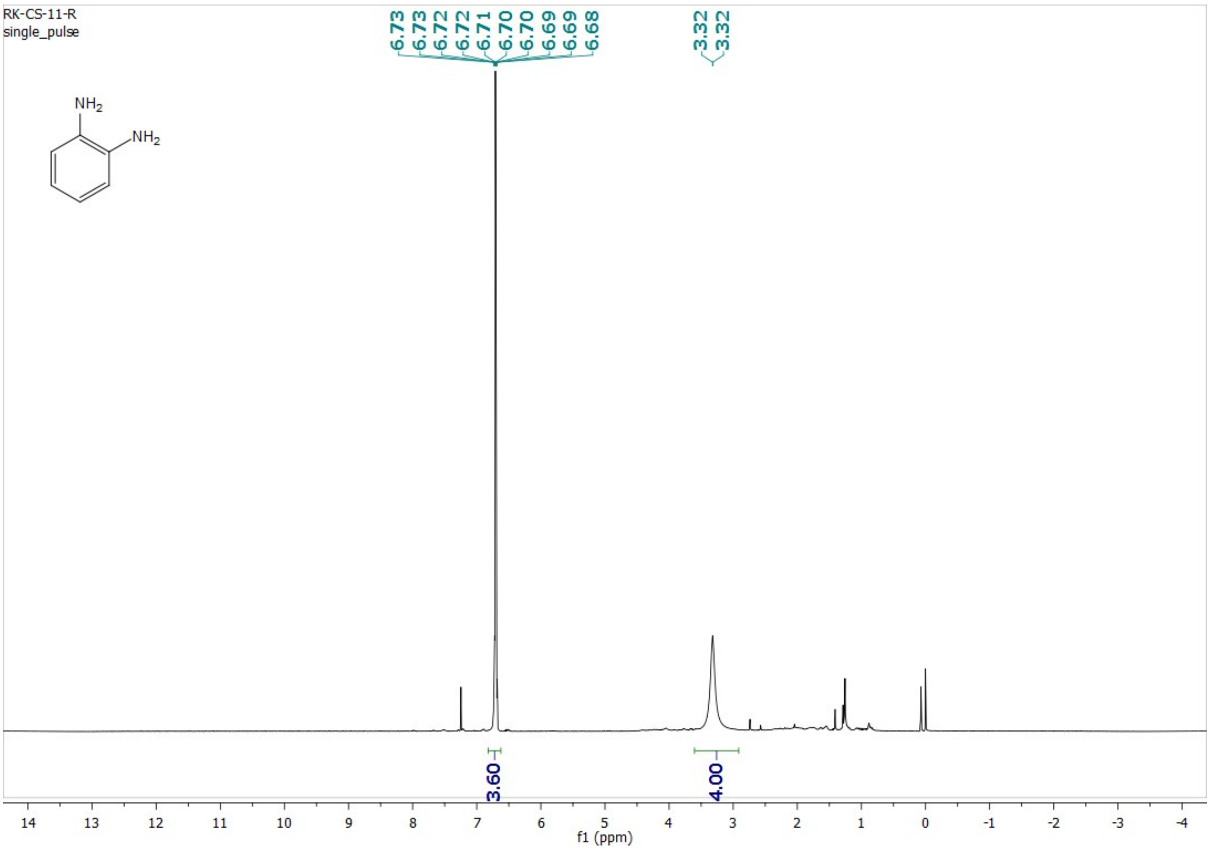
RK-CS-11-R
single_pulse
NH2
NH2
6.73
6.73
6.72
6.72
6.71
6.70
6.70
6.69
6.69
6.68
3.32
3.32
3.60
4.00
14 13 12 11 10 9 8 7 6 5 4 3 2 1 0 -1 -2 -3 -4
f1 (ppm)

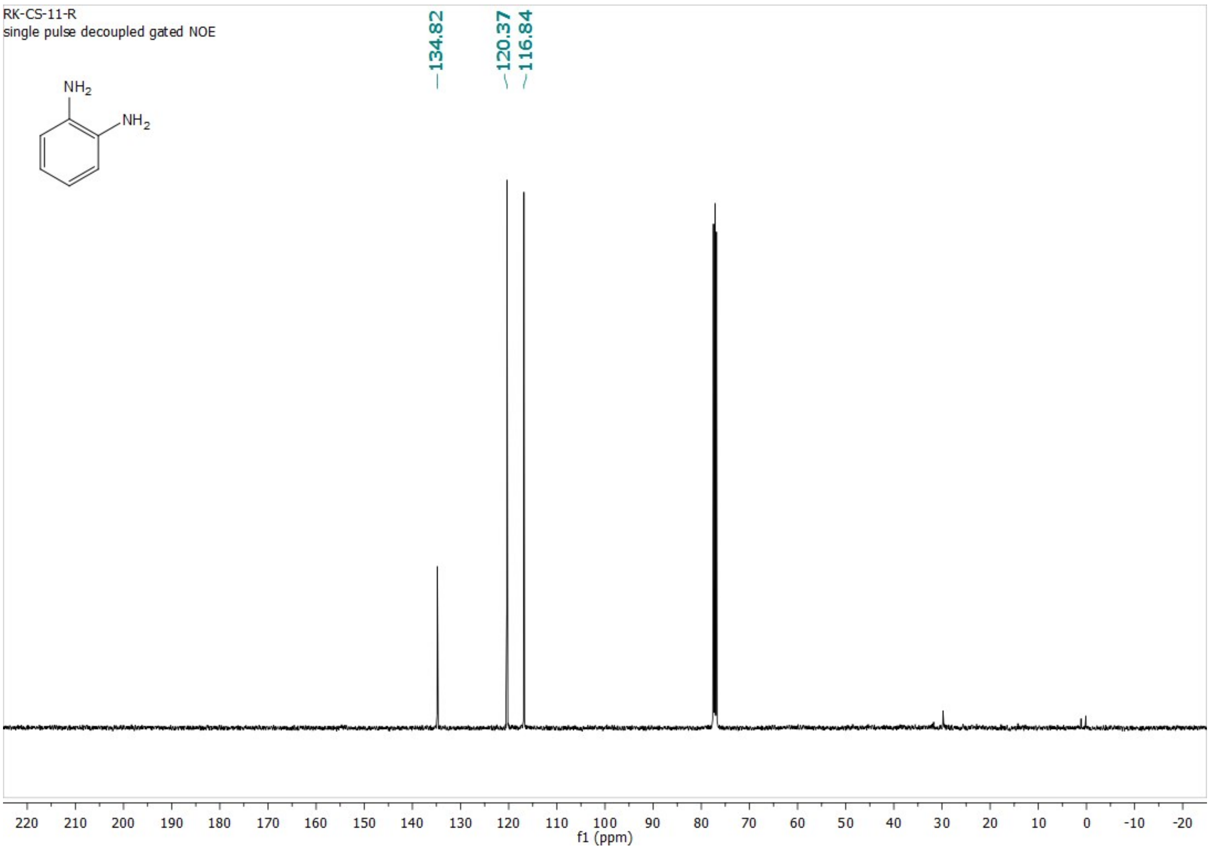
RK-CS-11-R
single pulse decoupled gated NOE
NH2
NH2
134.82
120.37
116.84
220 210 200 190 180 170 160 150 140 130 120 110 100 90 80 70 60 50 40 30 20 10 0 -10 -20
f1 (ppm)

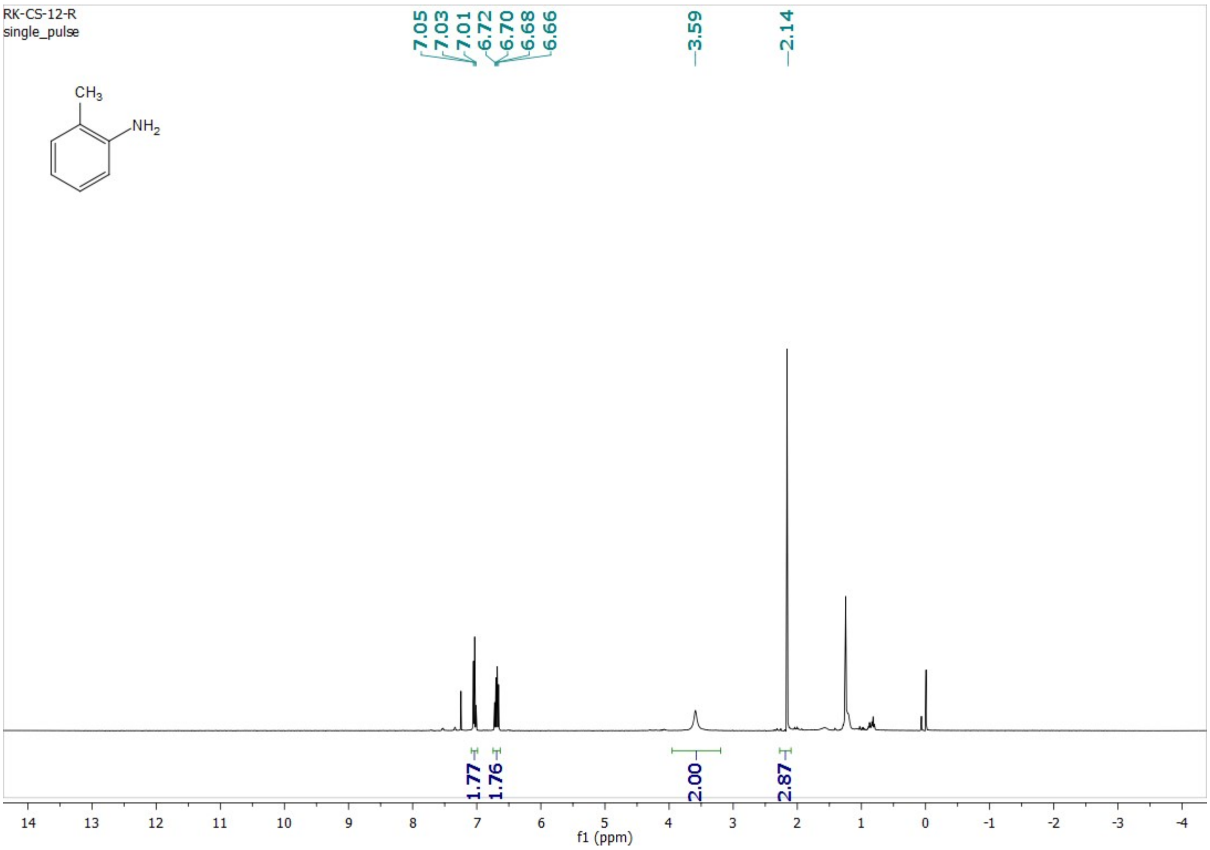

RK-CS-12-R
single_pulse
CH3
NH2
7.05
7.03
7.01
6.72
6.70
6.68
6.66
3.59
2.14
1.77
1.76
2.00
2.87
14 13 12 11 10 9 8 7 6 5 4 3 2 1 0 -1 -2 -3 -4
f1 (ppm)

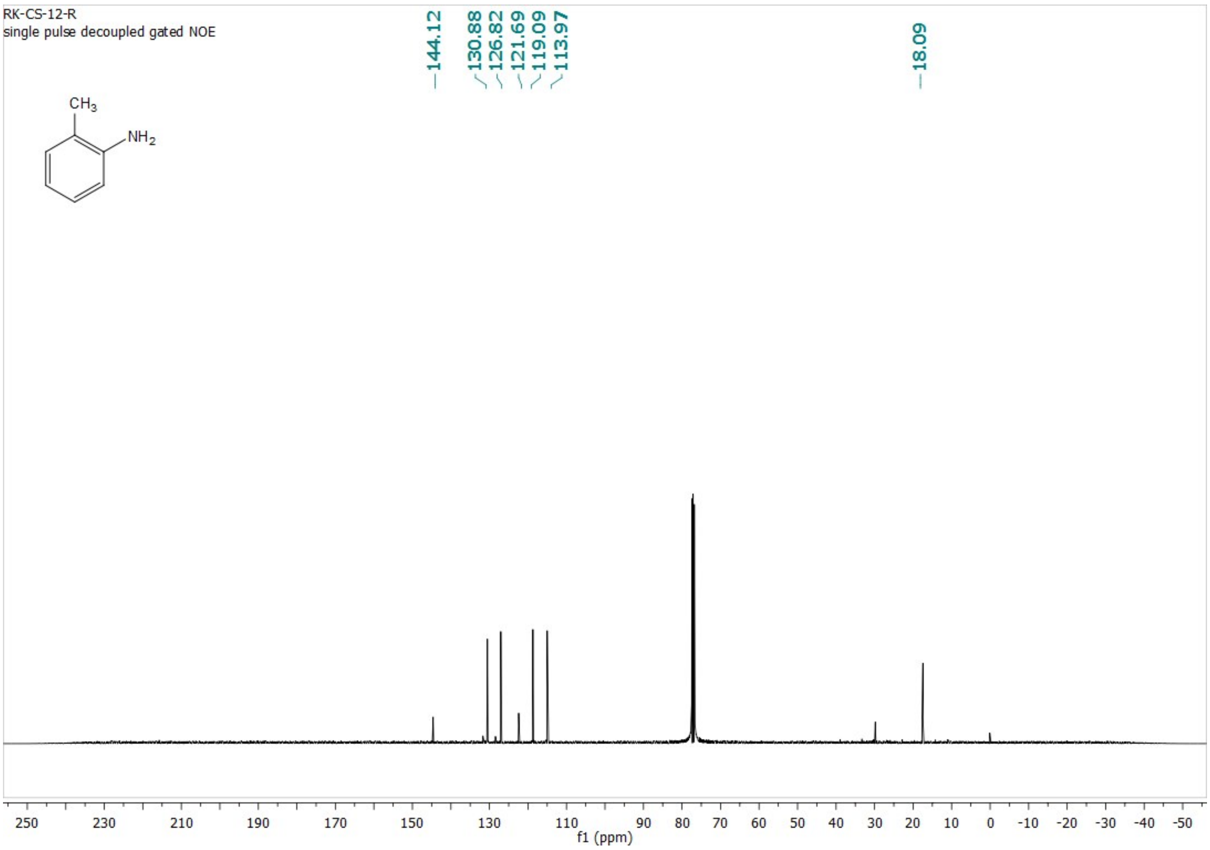

RK-CS-12-R
single pulse decoupled gated NOE
CH3
NH2
144.12
130.88
126.82
121.69
119.09
113.97
18.09
250 230 210 190 170 150 130 110 90 80 70 60 50 40 30 20 10 0 -10 -20 -30 -40 -50
f1 (ppm)

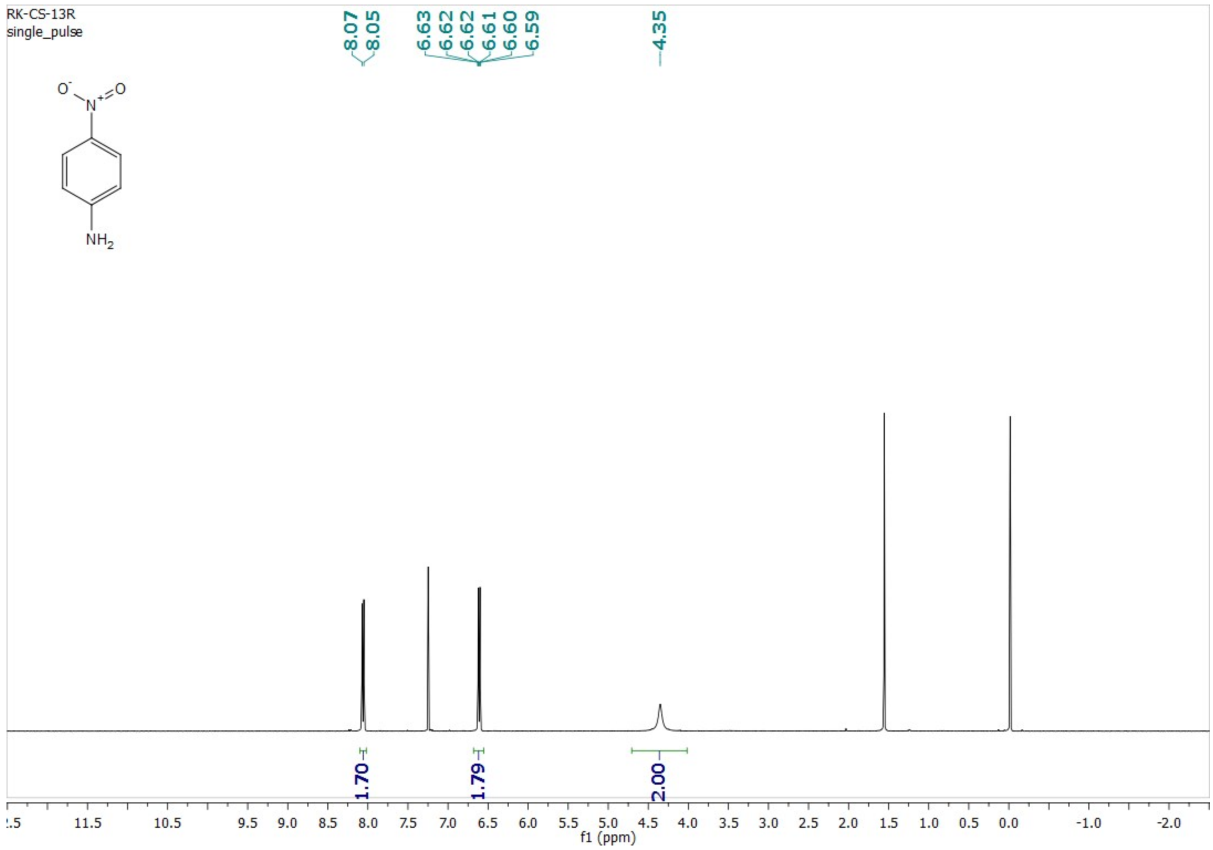
RK-CS-13R
single_pulse
8.07
8.05
6.63
6.62
6.62
6.61
6.60
6.59
4.35
1.70
1.79
2.00
f1 (ppm)

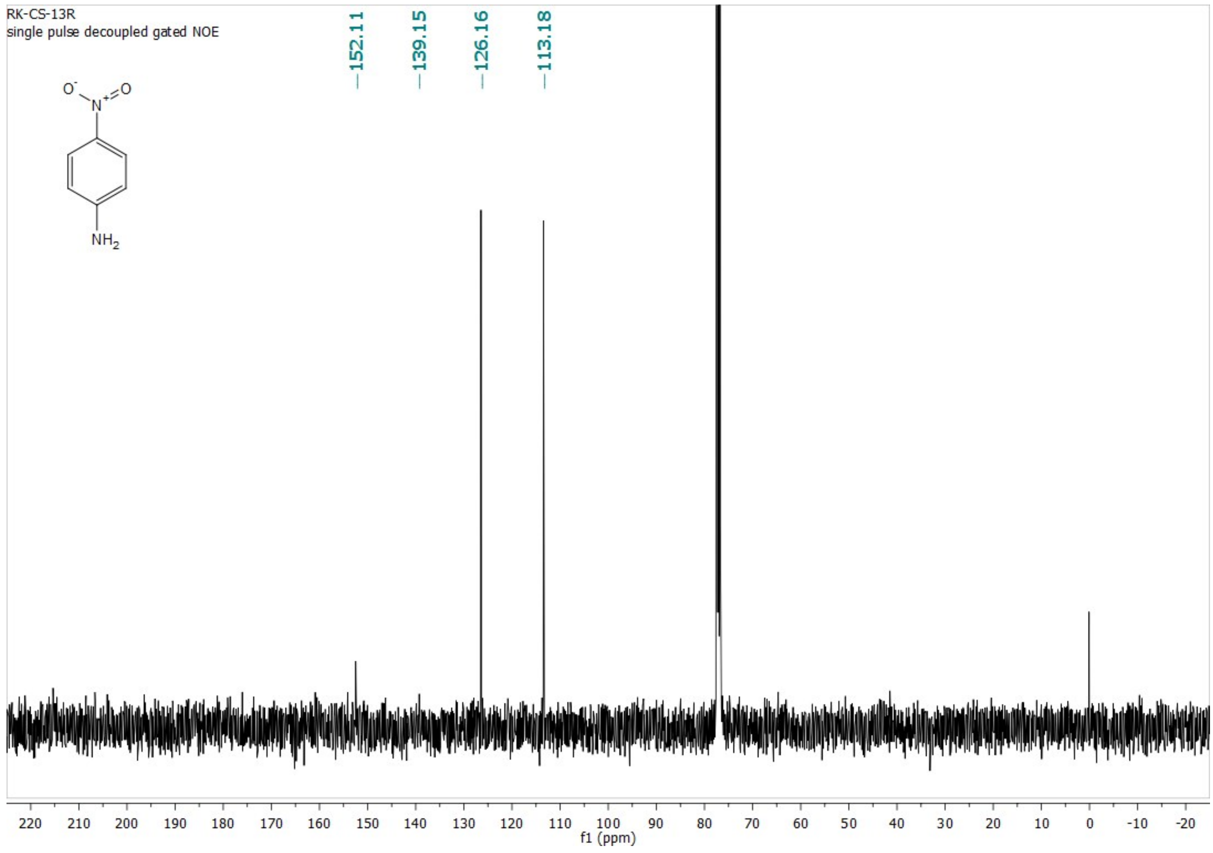
RK-CS-13R
single pulse decoupled gated NOE
152.11
139.15
126.16
113.18
f1 (ppm)

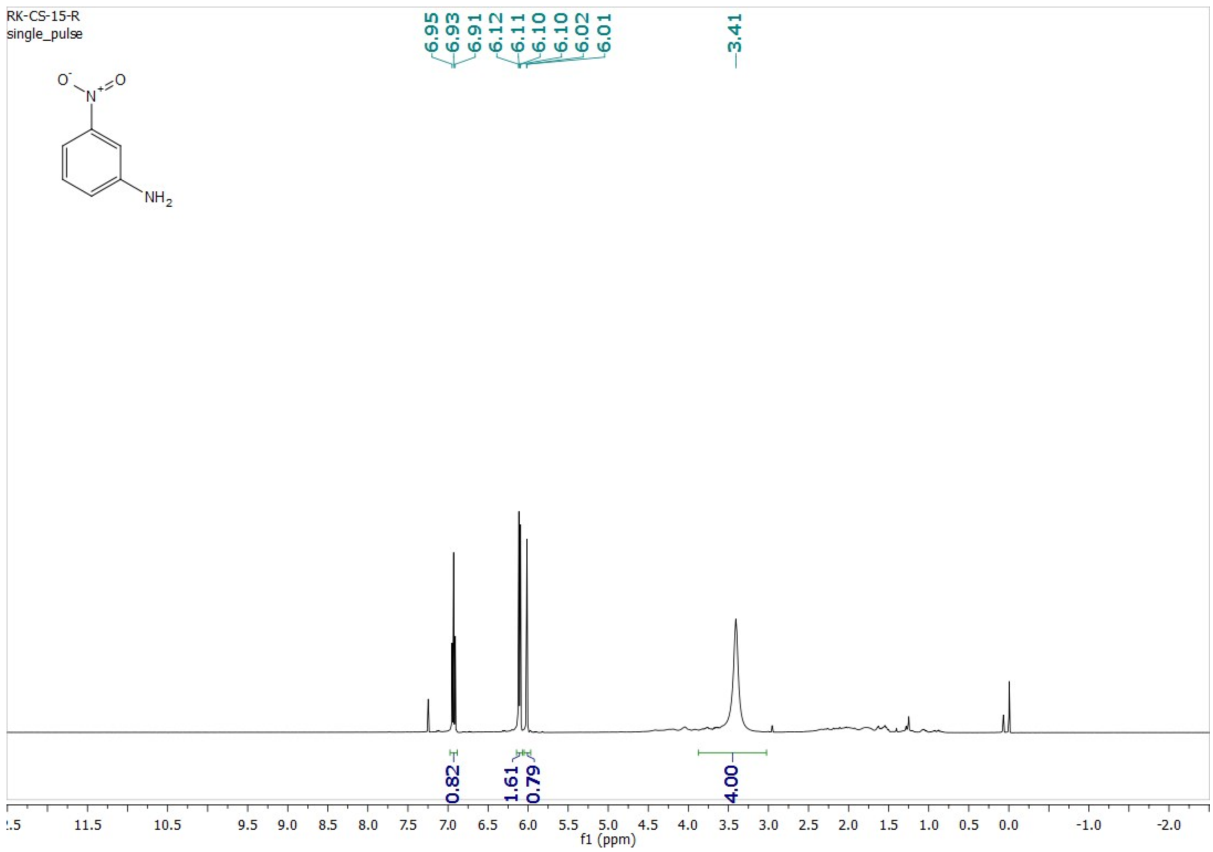
RK-CS-15-R
single_pulse
NH2
6.95
6.93
6.91
6.12
6.11
6.10
6.10
6.02
6.01
3.41
0.82
1.61
0.79
4.00
f1 (ppm)

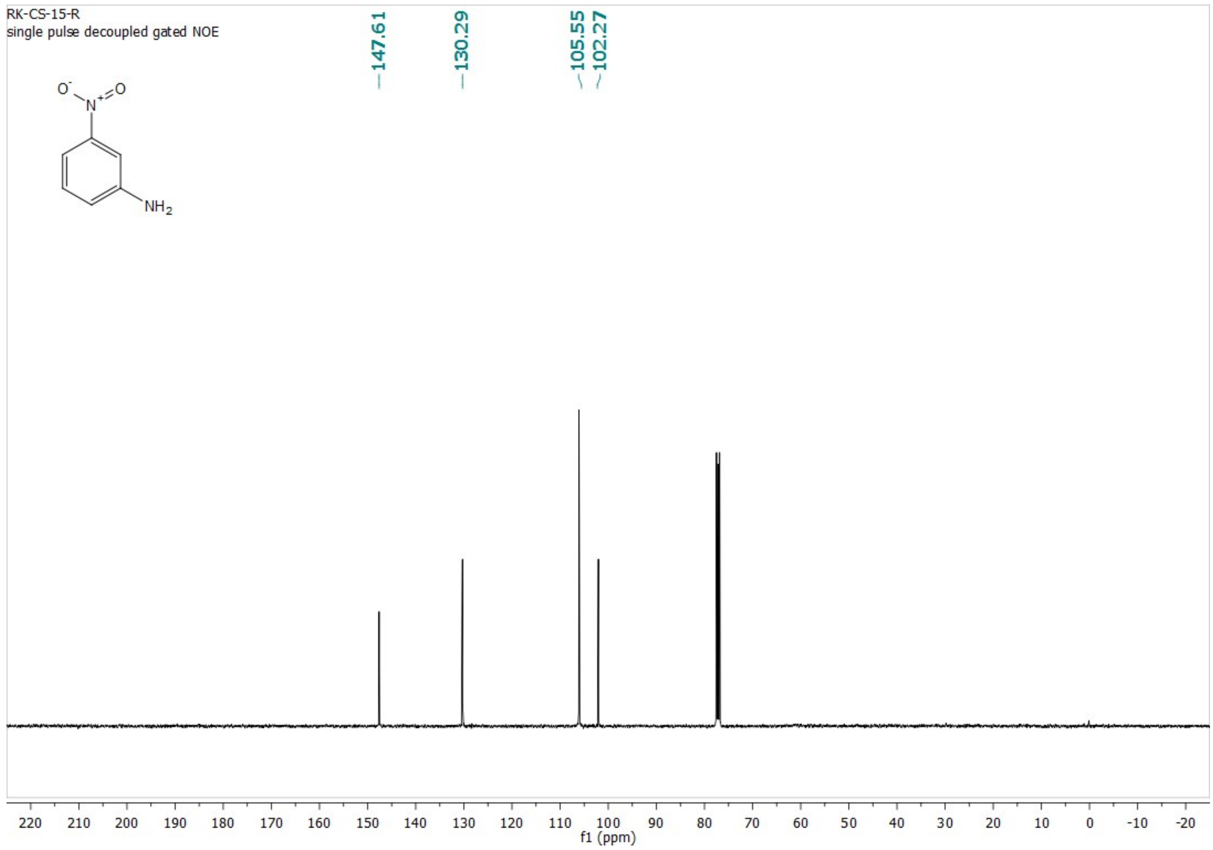
RK-CS-15-R
single pulse decoupled gated NOE
NH2
147.61
130.29
105.55
102.27
f1 (ppm)

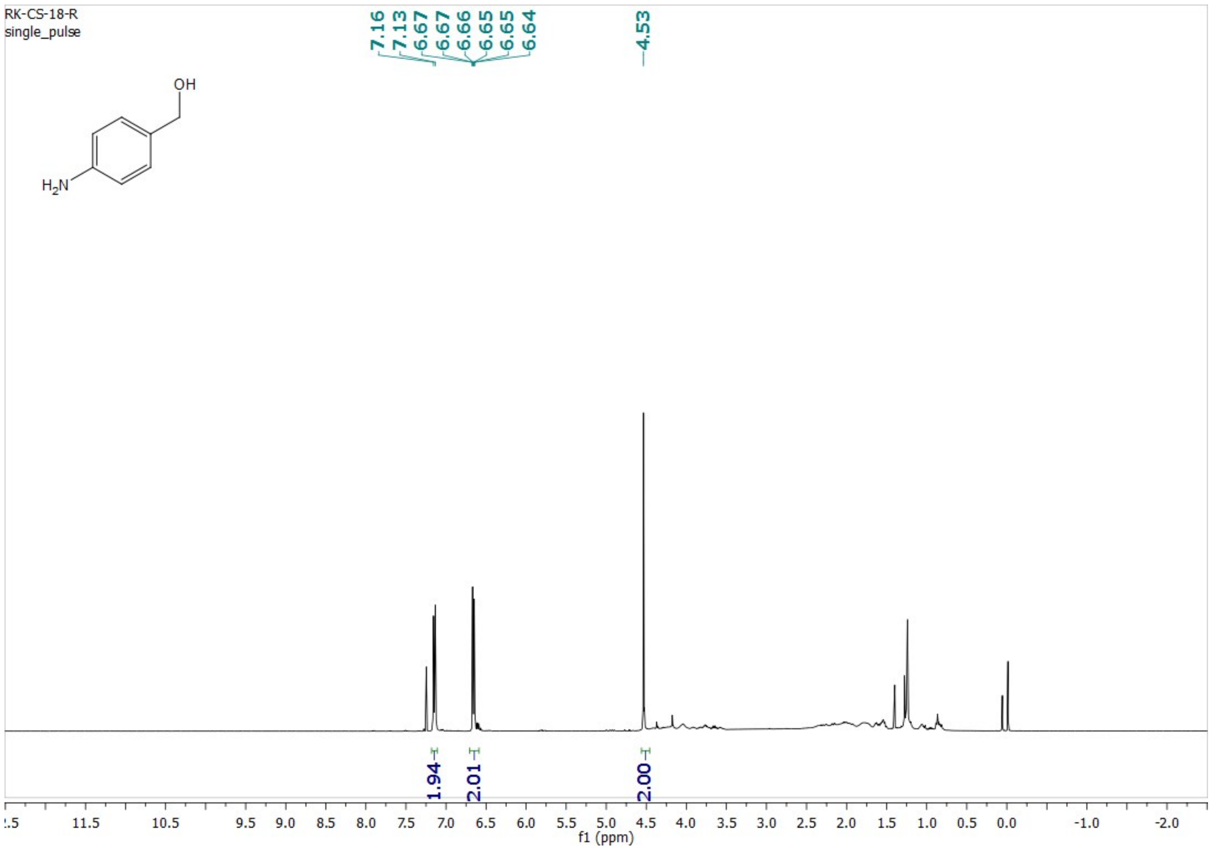
RK-CS-18-R
single_pulse
OH
H2N
7.16
7.13
6.67
6.67
6.66
6.65
6.65
6.64
4.53
1.94
2.01
2.00
f1 (ppm)

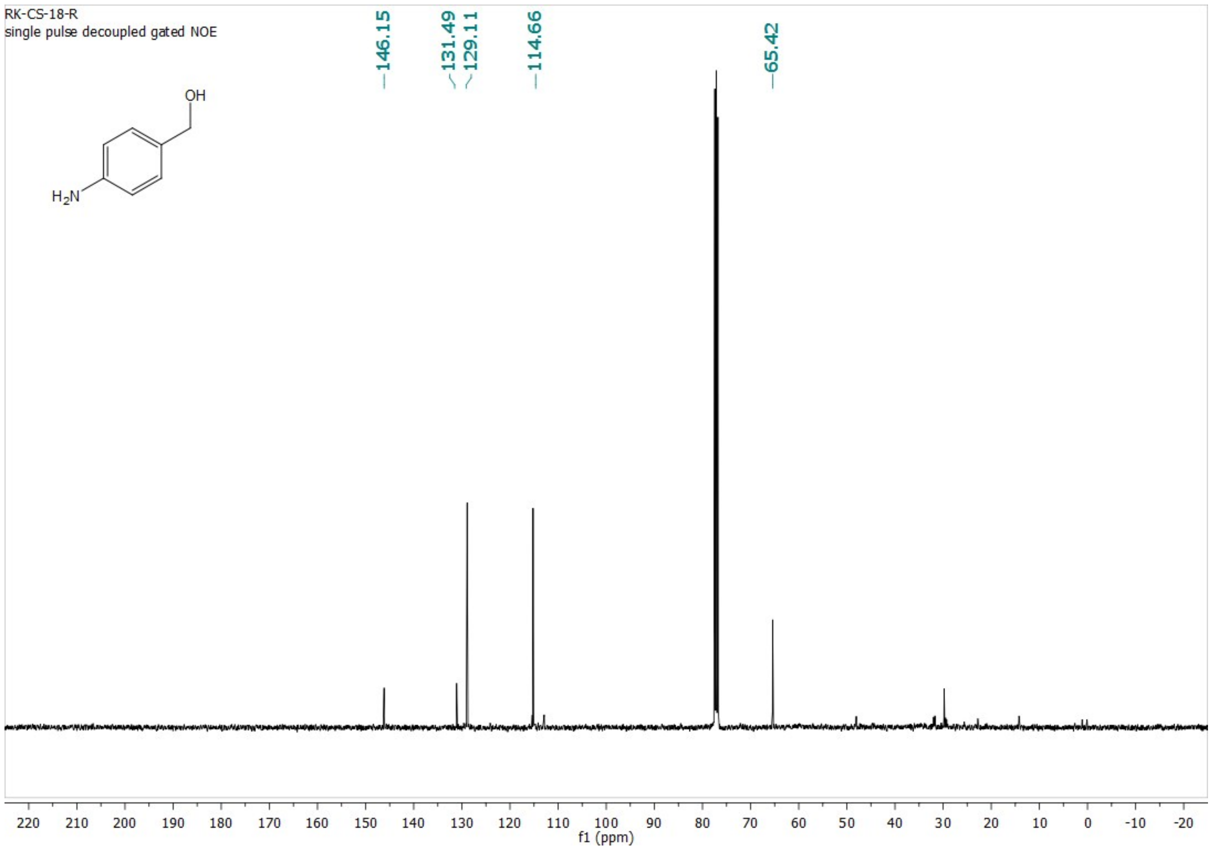
RK-CS-18-R
single pulse decoupled gated NOE
OH
H2N
146.15
131.49
129.11
114.66
65.42
f1 (ppm)

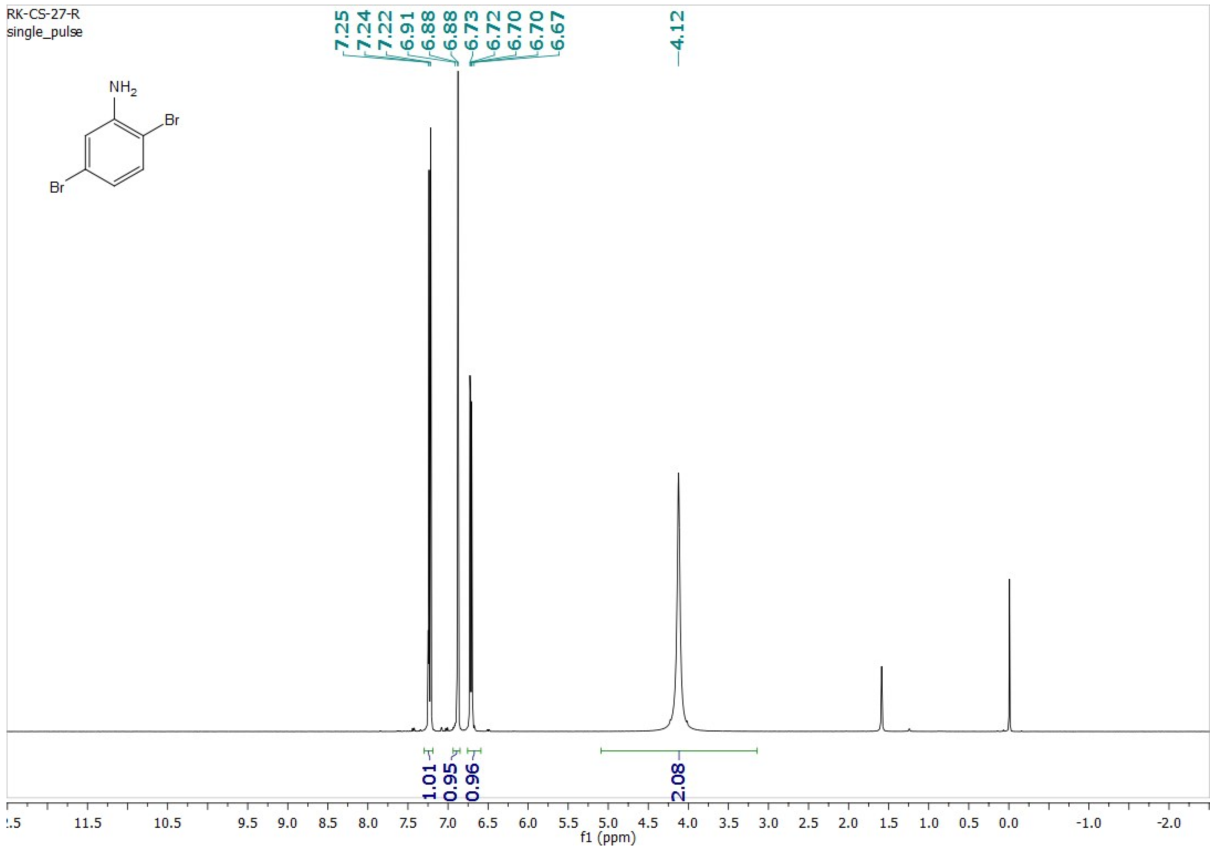
RK-CS-27-R
single_pulse
NH2
Br
Br
7.25
7.24
7.22
6.91
6.88
6.88
6.73
6.72
6.70
6.70
6.67
4.12
1.01
0.95
0.96
2.08
f1 (ppm)

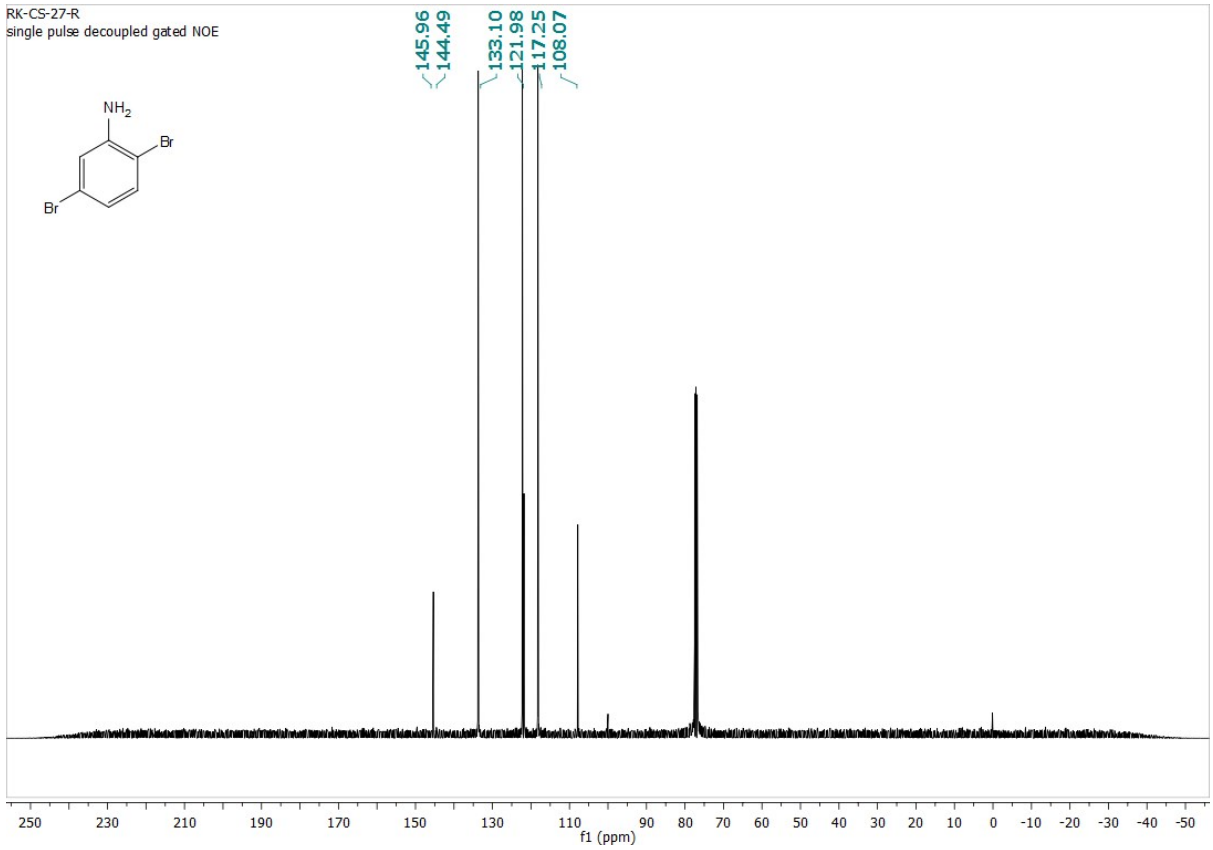
RK-CS-27-R
single pulse decoupled gated NOE
NH2
Br
Br
145.96
144.49
133.10
121.98
117.25
108.07
f1 (ppm)

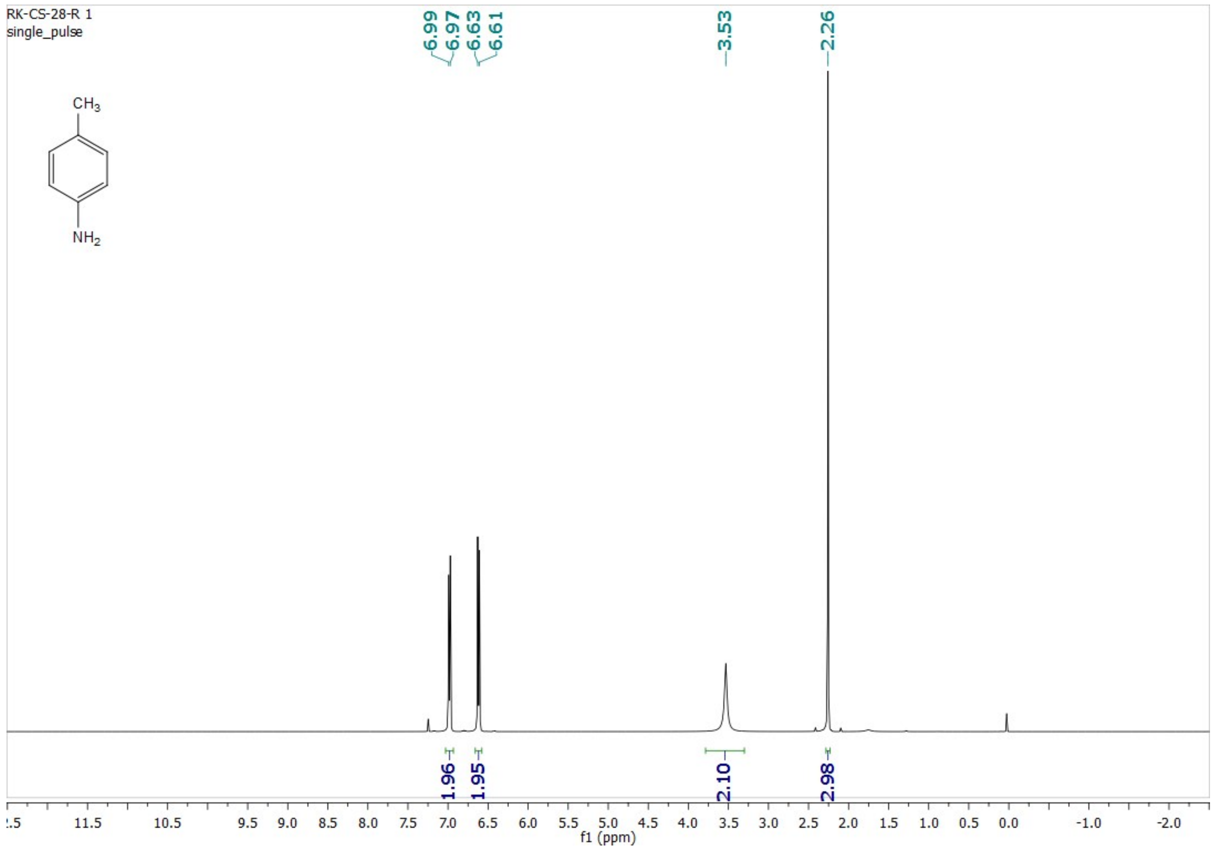
RK-CS-28-R 1
single_pulse
CH3
NH2
6.99
6.97
6.63
6.61
3.53
2.26
1.96
1.95
2.10
2.98
f1 (ppm)

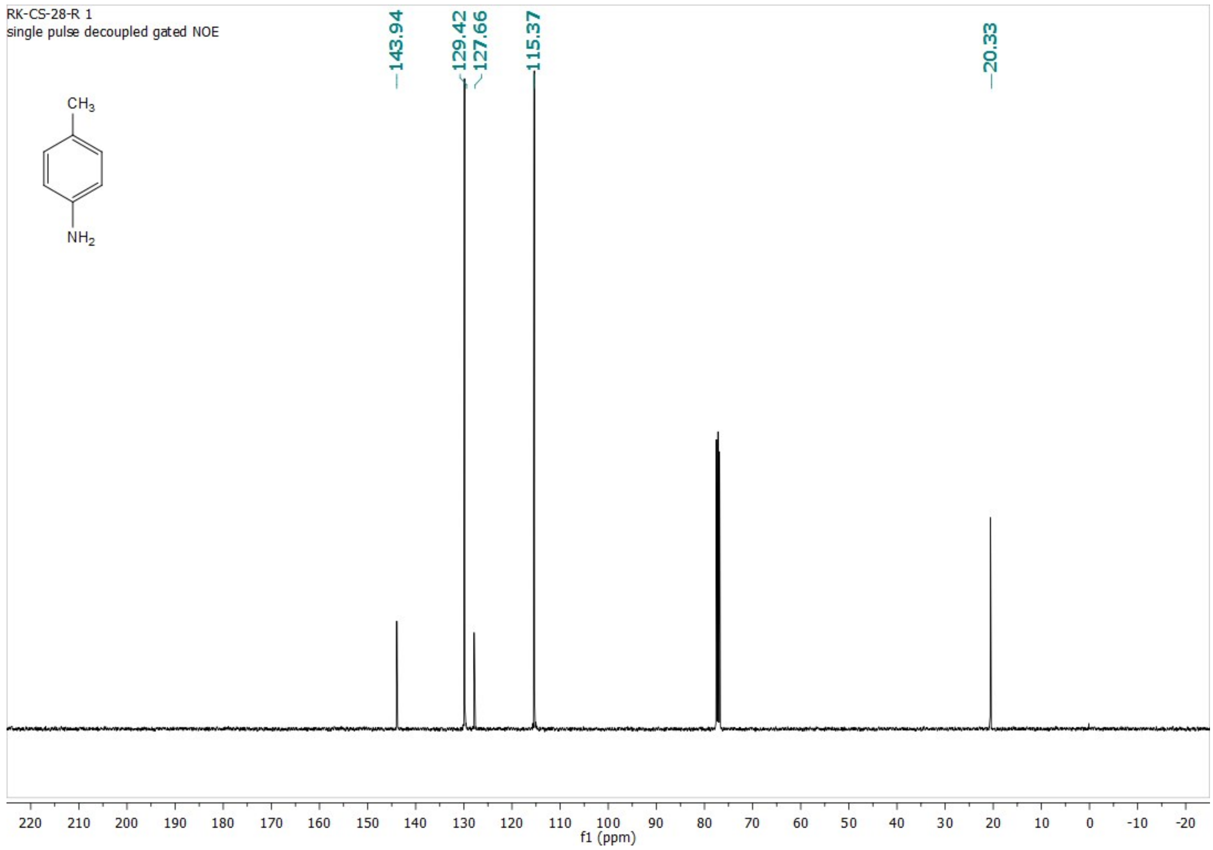
RK-CS-28-R 1
single pulse decoupled gated NOE
CH3
NH2
143.94
129.42
127.66
115.37
20.33
f1 (ppm)

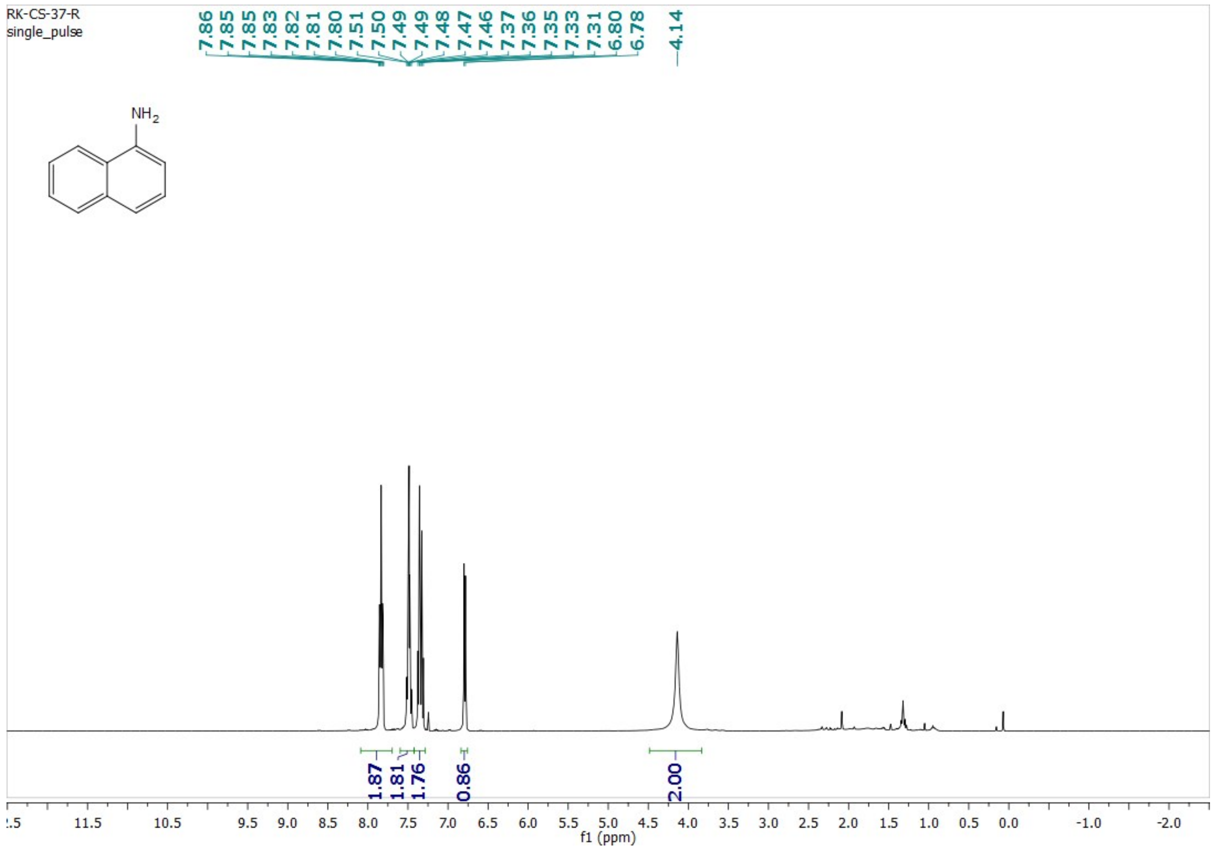
RK-CS-37-R
single_pulse
NH2
7.86 7.85 7.85 7.83 7.82 7.81 7.80 7.51 7.50 7.49 7.49 7.48 7.47 7.46 7.37 7.36 7.35 7.33 7.31 6.80 6.78
4.14
1.87
1.81
1.76
0.86
2.00
f1 (ppm)

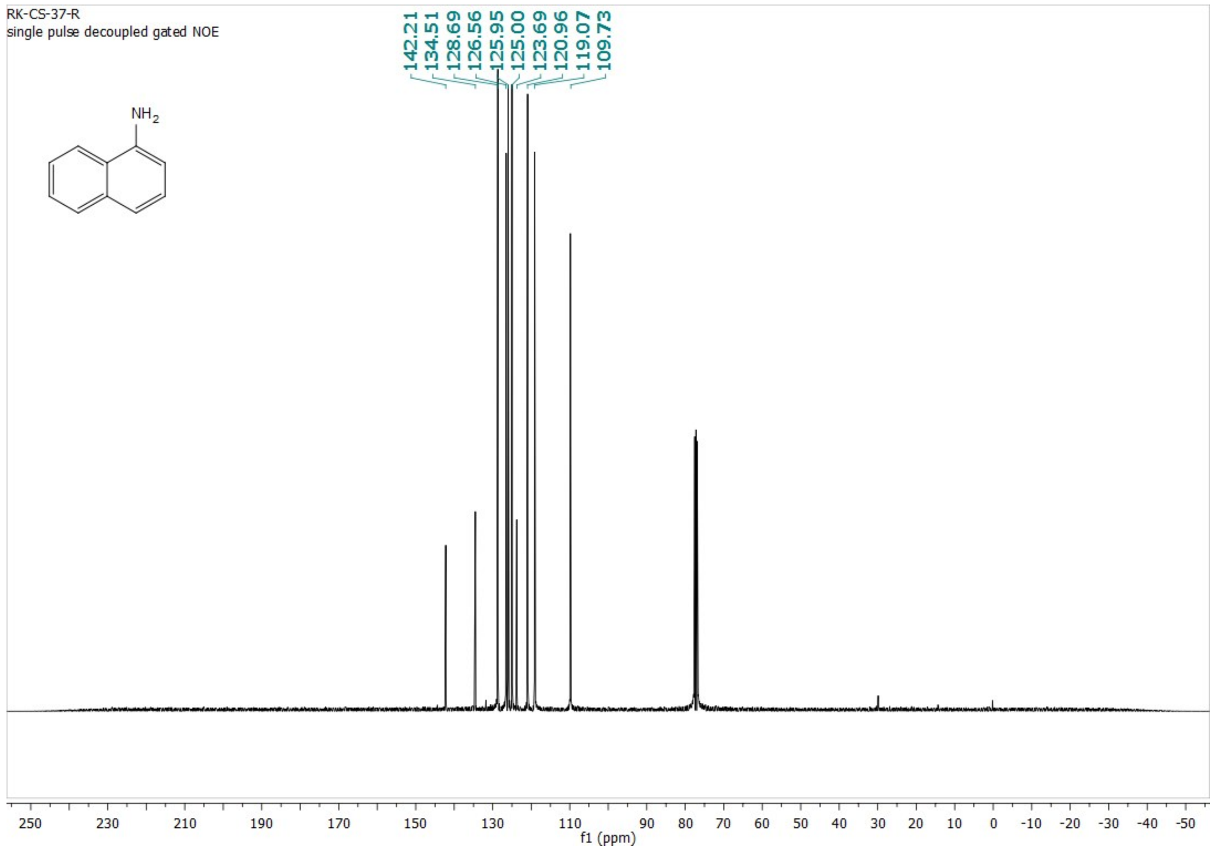
RK-CS-37-R
single pulse decoupled gated NOE
NH2
142.21 134.51 128.69 126.56 125.95 125.00 123.69 120.96 119.07 109.73
f1 (ppm)

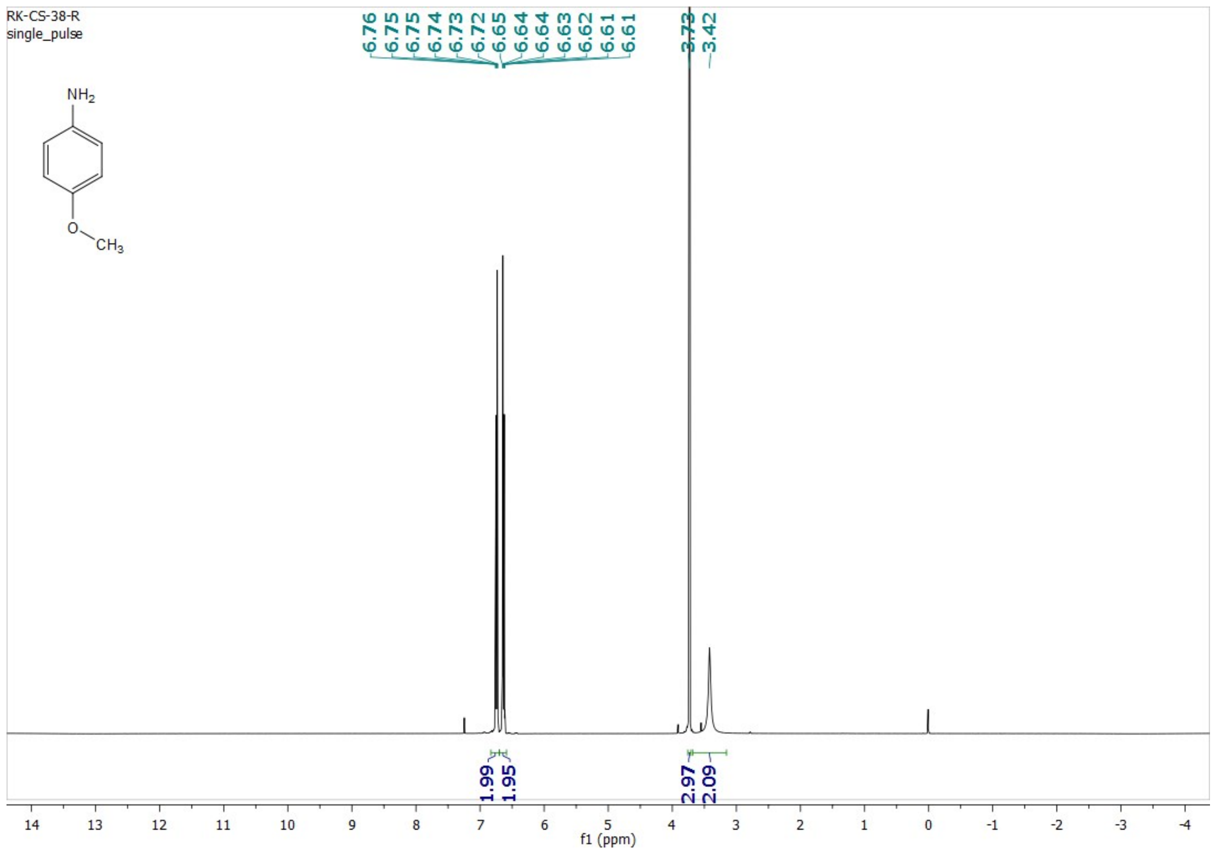
RK-CS-38-R
single_pulse
NH2
O
CH3
6.76
6.75
6.75
6.74
6.73
6.72
6.65
6.64
6.64
6.63
6.62
6.61
6.61
3.73
3.42
1.99
1.95
2.97
2.09
f1 (ppm)

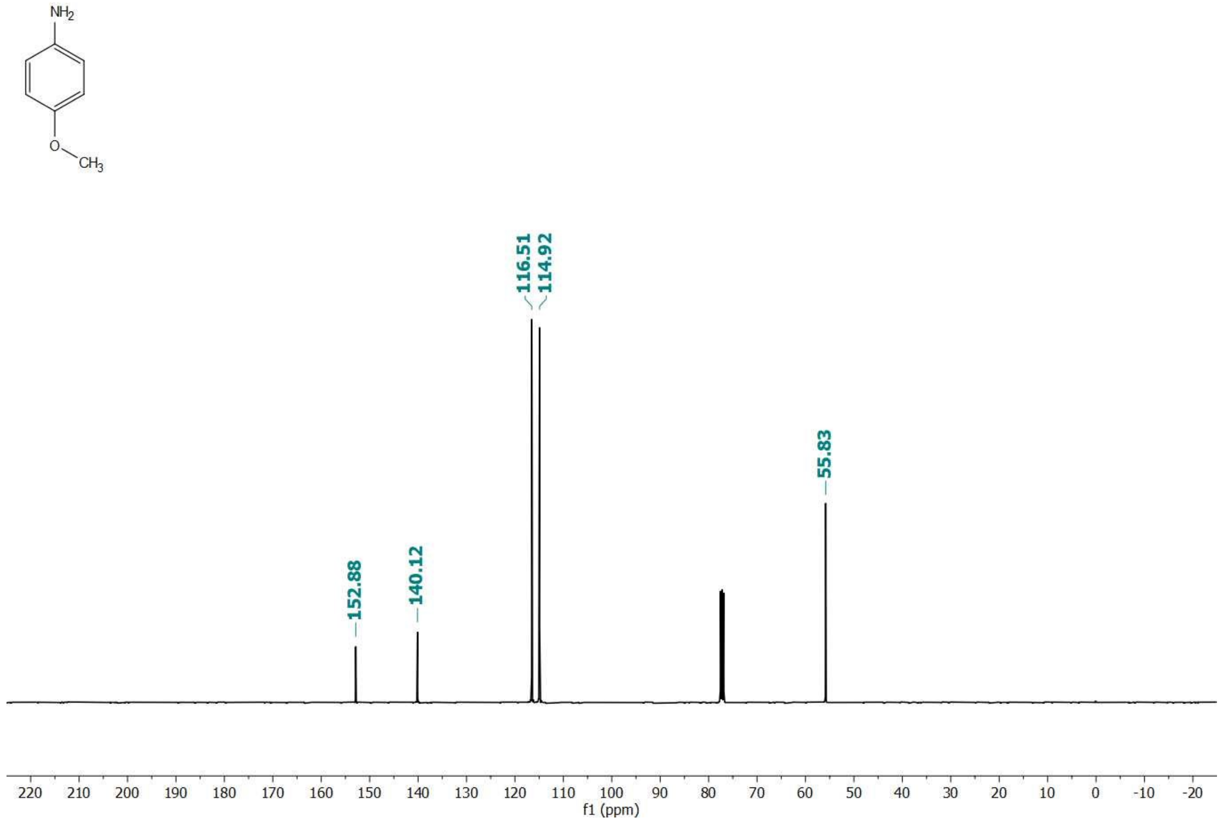
NH2
O
CH3
116.51
114.92
55.83
152.88
140.12
f1 (ppm)

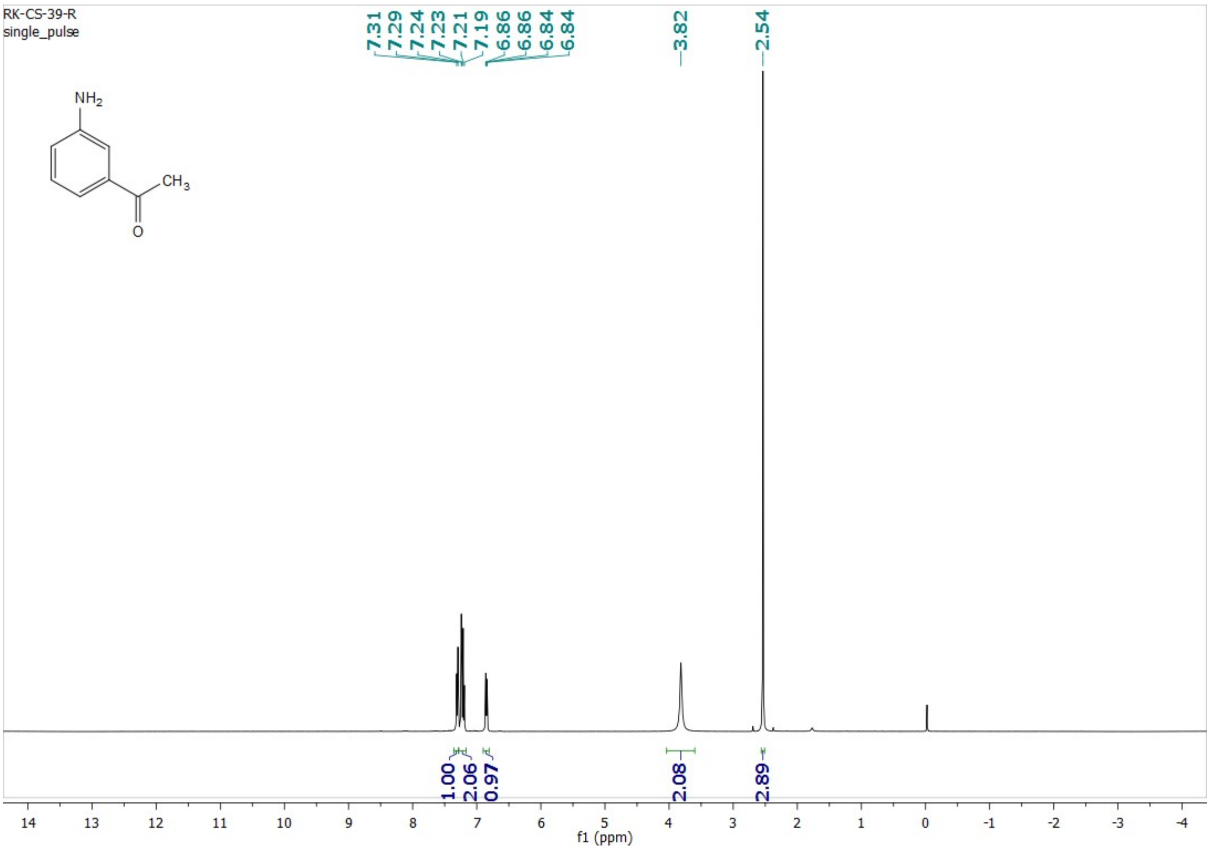
RK-CS-39-R
single_pulse
7.31
7.29
7.24
7.23
7.21
7.19
6.86
6.86
6.84
6.84
3.82
2.54
NH2
CH3
O
1.00
2.06
0.97
2.08
2.89
14
13
12
11
10
9
8
7
6
5
4
3
2
1
0
-1
-2
-3
-4
f1 (ppm)

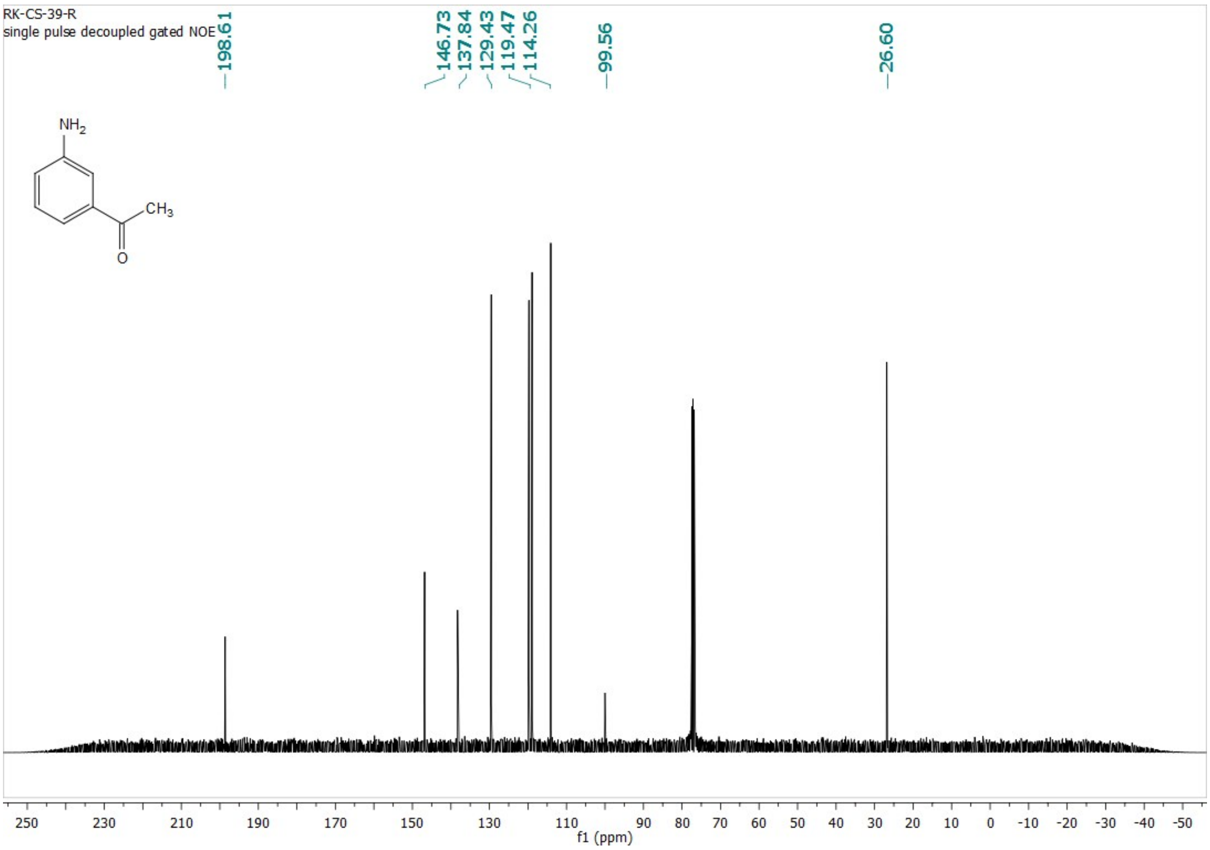
RK-CS-39-R
single pulse decoupled gated NOE
198.61
146.73
137.84
129.43
119.47
114.26
99.56
26.60
NH2
CH3
O
250
230
210
190
170
150
130
110
90
80
70
60
50
40
30
20
10
0
-10
-20
-30
-40
-50
f1 (ppm)

References

1. D. Cantillo, M. M. Moghaddam, C. O. Kappe, *Journal of Organic Chemistry*, **2013**, 78, 4530-4542
2. N. R. Lee, A. A. Bikovtseva, M. C. Clerget, F. Gallou, B. H. Lipshutz, *Organic Letters*, **2017**, 19, 6518-6521
3. R. Dey, N. Mukherjee, S. Ahammed, B. C. Ranu, *Chem Comm*, **2012**, 48, 7982-7984
4. K. Junge, B. Wendt, N. Shaikh, M. Beller, *Chem Comm*, **2010**, 46, 1769-1771
5. S. Kim, E. Kim, B. M. Kim, *Chemistry an Asian Journal*, **2011**, 6, 1921-1925
6. L. Pehlivan, E. Metay, S. Laval, W. Dayoub, P. Demonchaux, G. Mignani, M. Lemaire, *Tetrahedron Letters*, **51**, 1939-1941
7. P. S. Rathore, R. Patidar, T. Shripathi, S. Thakore, *Catalysis Science & Technology*, **2015**, 5, 286-295
8. R. K. Sharma, Y. Monga, A. Puri, *Journal of Molecular Catalysis*, **2014**, 393, 84-95
